# Novel insights into the relationships between dendritic cell subsets in human and mouse revealed by genome-wide expression profiling

**DOI:** 10.1186/gb-2008-9-1-r17

**Published:** 2008-01-24

**Authors:** Scott H Robbins, Thierry Walzer, Doulaye Dembélé, Christelle Thibault, Axel Defays, Gilles Bessou, Huichun Xu, Eric Vivier, MacLean Sellars, Philippe Pierre, Franck R Sharp, Susan Chan, Philippe Kastner, Marc Dalod

**Affiliations:** 1 CIML (Centre d'Immunologie de Marseille-Luminy), Université de la Méditerranée, Parc scientifique de Luminy case 906, Marseille F-13288, France; 2 U631, INSERM (Institut National de la Santé et de la Recherche Médicale), Parc scientifique de Luminy case 906, Marseille F-13288, France; 3 UMR6102, CNRS (Centre National de la Recherche Scientifique), Parc scientifique de Luminy case 906, Marseille F-13288, France; 4 Hematopoiesis and leukemogenesis in the mouse, IGBMC (Institut de Génétique et de Biologie Moléculaire et Cellulaire), rue Laurent Fries, ILLKIRCH F-67400, France; 5 U596, INSERM, rue Laurent Fries, ILLKIRCH F-67400, France; 6 UMR7104, CNRS, rue Laurent Fries, ILLKIRCH F-67400, France; 7 UM41, Université Louis Pasteur, rue Laurent Fries, Strasbourg F-67400, France; 8 The Medical Investigation of Neurodevelopmental Disorders Institute, University of California at Davis Medical Center, Sacramento, CA 95817, USA; 9 Hôpital de la Conception, Assistance Publique-Hôpitaux de Marseille, Boulevard Baille, Marseille F-13385, France; 10 Current address: Genomics Institute of the Novartis Research Foundation, John Jay Hopkins Drive, San Diego, CA 92121, USA

## Abstract

**Background:**

Dendritic cells (DCs) are a complex group of cells that play a critical role in vertebrate immunity. Lymph-node resident DCs (LN-DCs) are subdivided into conventional DC (cDC) subsets (CD11b and CD8α in mouse; BDCA1 and BDCA3 in human) and plasmacytoid DCs (pDCs). It is currently unclear if these various DC populations belong to a unique hematopoietic lineage and if the subsets identified in the mouse and human systems are evolutionary homologs. To gain novel insights into these questions, we sought conserved genetic signatures for LN-DCs and *in vitro *derived granulocyte-macrophage colony stimulating factor (GM-CSF) DCs through the analysis of a compendium of genome-wide expression profiles of mouse or human leukocytes.

**Results:**

We show through clustering analysis that all LN-DC subsets form a distinct branch within the leukocyte family tree, and reveal a transcriptomal signature evolutionarily conserved in all LN-DC subsets. Moreover, we identify a large gene expression program shared between mouse and human pDCs, and smaller conserved profiles shared between mouse and human LN-cDC subsets. Importantly, most of these genes have not been previously associated with DC function and many have unknown functions. Finally, we use compendium analysis to re-evaluate the classification of interferon-producing killer DCs, lin^-^CD16^+^HLA-DR^+ ^cells and *in vitro *derived GM-CSF DCs, and show that these cells are more closely linked to natural killer and myeloid cells, respectively.

**Conclusion:**

Our study provides a unique database resource for future investigation of the evolutionarily conserved molecular pathways governing the ontogeny and functions of leukocyte subsets, especially DCs.

## Abstract 2

Genome-wide expression profiling of mouse and human leukocytes reveal conserved transcriptional programs of plasmacytoid or conventional dendritic cell subsets.

## Background

Dendritic cells (DCs) were initially identified by their unique ability to present antigen for the priming of naïve CD4 and CD8 T lymphocytes [1]. DCs have more recently been shown to be key sentinel immune cells able to sense, and respond to, danger very early in the course of an infection due to their expression of a broad array of pattern recognition receptors [2]. Indeed, DCs have been shown to play a major role in the early production of effector antimicrobial molecules such as interferon (IFN)-α and IFN-β [3] or inducible nitric oxide synthase [4] and it has been demonstrated that DCs can also activate other innate effector cells such as natural killer (NK) cells [5]. In light of these properties, it has been clearly established that DCs are critical for defense against infections, as they are specially suited for the early detection of pathogens, the rapid development of effector functions, and the triggering of downstream responses in other innate and adaptive immune cells.

DCs can be divided into several subsets that differ in their tissue distribution, their phenotype, their functions and their ontogeny [6]. Lymph node-resident DCs (LN-DCs) encompass conventional DCs (cDCs) and plasmacytoid DCs (pDCs) in both humans and mice. LN-cDCs can be subdivided into two populations in both mouse (CD8α and CD11b cDCs) [6] and in human (BDCA1 and BDCA3 cDCs) [7]. In mouse, CD8α cDCs express many scavenger receptors and may be especially efficient for cross-presenting antigen to CD8 T cells [8] whereas CD11b cDCs have been suggested [9,10], and recently shown [11], to be specialized in the activation of CD4 T cells. As human cDC functions are generally studied with cells derived *in vitro *from monocytes or from CD34^+ ^hematopoietic progenitors, which may differ considerably from the naturally occurring DCs present *in vivo*, much less is known of the eventual functional specialization of human cDC subsets. Due to differences in the markers used for identifying DC subsets between human and mouse and to differences in the expression of pattern recognition receptors between DC subsets, it has been extremely difficult to address whether there are functional equivalences between mouse and human cDC subsets [6].

pDCs, a cell type discovered recently in both human and mouse, appear broadly different from the other DC subsets to the point that their place within the DC family is debated [3]. Some common characteristics between human and mouse pDCs that distinguish them from cDCs [3] include: their ability to produce very large amounts of IFN-α/β upon activation, their limited ability to prime naïve CD4 and CD8 T cells under steady state conditions, and their expression of several genes generally associated with the lymphocyte lineage and not found in cDCs [12]. Several differences have also been reported between human and mouse pDCs, which include the unique ability of mouse pDCs to produce high levels of IL-12 upon triggering of various toll-like receptors (TLRs) or stimulation with viruses [13,14]. Adding to the complexity of accurately classifying pDCs within leukocyte subsets are recent reports describing cell types bearing mixed phenotypic and functional characteristics of NK cells and pDCs in the mouse [15,16]. Collectively, these findings raise the question of how closely related human and mouse pDCs are to one another or to cDCs as compared to other leukocyte populations.

Global transcriptomic analysis has recently been shown to be a powerful approach to yield new insights into the biology of specific cellular subsets or tissues through their specific gene expression programs [17-21]. Likewise, genome-wide comparative gene expression profiling between mouse and man has recently been demonstrated as a powerful approach to uncover conserved molecular pathways involved in the development of various cancers [22-27]. However, to the best of our knowledge, this approach has not yet been applied to study normal leukocyte subsets. Moreover, DC subsets have not yet been scrutinized through the prism of gene expression patterns within the context of other leukocyte populations. In this report, we assembled compendia comprising various DC and other leukocyte subtypes, both from mouse and man. Using intra- and inter-species comparisons, we define the common and specific core genetic programs of DC subsets.

## Results

### Generation/assembly and validation of the datasets for the gene expression profiling of LN-DC subsets

We used pan-genomic Affymetrix Mouse Genome 430 2.0 arrays to generate gene expression profiles of murine splenic CD8α (n = 2) and CD11b (n = 2) cDCs, pDCs (n = 2), B cells (n = 3), NK cells (n = 2), and CD8 T cells (n = 2). To generate a compendium of 18 mouse leukocyte profiles, these data were complemented with published data retrieved from public databases, for conventional CD4 T cells (n = 2) [28] and splenic macrophages (n = 3) [29]. We used Affymetrix Human Genome U133 Plus 2.0 arrays to generate gene expression profiles of blood monocytes, neutrophils, B cells, NK cells, and CD4 or CD8 T cells [30]. These data were complemented with published data on human blood DC subsets (pDCs, BDCA1 cDCs, BDCA3 cDCs, and lin^-^CD16^+^HLA-DR^+ ^cells) retrieved from public databases [31]. All of the human samples were done in independent triplicates. Information regarding the original sources and the public accessibility of the datasets analyzed in the paper are given in Table 1.

**Table 1:** Information on the sources and public access for the datasets analyzed in the paper

					Figures^‡^							
					
Dataset	Population*	Laboratory^†^	Public repository	Accession number	1a,c; 2a	1b,d; 2b	1e	3	4a	4b	5a	5b
Affymetrix Mouse Genome 430 2.0 data												
	Spleen CD8 DCs (2)	MD/SCPK	GEO [95]	GSE9810	X		X	X	X		X	
	Spleen CD11b DCs (2)	MD/SCPK	GEO	GSE9810	X		X	X	X		X	
	Spleen pDCs (2)	MD/SCPK	GEO	GSE9810	X		X	X	X		X	
	Spleen NK cells (2)	MD/SCPK	GEO	GSE9810	X		X		X			
	Spleen CD8 T cells (2)	MD/SCPK	GEO	GSE9810	X		X					
	Spleen B cells (3)	MD/SCPK	GEO	GSE9810	X		X		X			
	Spleen CD4 T cells (2)	AYR	GEO	GSM44979; GSM44982	X		X		X			
	Spleen monocytes (3)	SB	NCI caArray [96]	NA	X		X				X	
	Spleen monocytes (2)	BP	GEO	GSM224733; GSM224735							X	
	Peritoneal MΦ (1)	SA	GEO	GSM218300							X	
	BM-MΦ (2)	RM	GEO	GSM177078; GSM177081							X	
	BM-MΦ (1)	CK	GEO	GSM232005							X	
	BM-DCs (2)	RM	GEO	GSM40053; GSM40056							X	
	BM-DCs (2)	MH	GEO	GSM101418; GSM101419							X	
Affymetrix Mouse U74Av2 data												
	Spleen CD4 T cells (3)	CB/DM	GEO	GSM66901; GSM66902; GSM66903					X			
	Spleen B2 cells (2)	CB/DM	GEO	GSM66913; GSM66914					X			
	Spleen B1 cells (2)	CB/DM	GEO	GSM66915; GSM66916					X			
	Spleen NK cells (2)	FT	EBI ArrayExpress [97]	E-MEXP-354					X			
	Spleen CD4 DCs (2)	CRES	GEO	GSM4697; GSM4707					X			
	Spleen CD8 DCs (2)	CRES	GEO	GSM4708; GSM4709					X			
	Spleen DN DCs (2)	CRES	GEO	GSM4710; GSM4711					X			
	Spleen IKDCs (2)	FH	GEO	GSM85329; GSM85330					X			
	Spleen cDCs (2)	FH	GEO	GSM85331; GSM85332					X			
	Spleen pDCs (2)	FH	GEO	GSM85333; GSM85334					X			
Affymetrix Human Genome U133 Plus 2.0 data												
	Blood monocytes (3)	FRS	Authors' webpage [86]	NA		X	X			X		X
	Blood CD4 T cells (3)	FRS	Authors' webpage	NA		X	X			X		
	Blood CD8 T cells (3)	FRS	Authors' webpage	NA		X	X			X		
	Blood B cells (3)	FRS	Authors' webpage	NA		X	X			X		
	Blood NK cells (3)	FRS	Authors' webpage	NA		X	X			X		
	Blood neutrophils (3)	FRS	Authors' webpage	NA		X	X			X		
	Blood pDCs (3)	CAKB	EBI ArrayExpress	E-TABM-34		X	X	X		X		X
	Blood BDCA1 DCs (3)	CAKB	EBI ArrayExpress	E-TABM-34		X	X	X		X		X
	Blood BDCA3 DCs (3)	CAKB	EBI ArrayExpress	E-TABM-34		X	X	X		X		X
	Blood CD16 DCs (3)	CAKB	EBI ArrayExpress	E-TABM-34						X		X
	PBMC-derived MΦ (2)	SYH	GEO	GSM109788; GSM109789								X
	Monocyte-derived MΦ	LZH	GEO	GSM213500								X
	Monocyte-derived DCs (3)	MVD	GEO	GSM181931; GSM181933; GSM181971								X

*The number of replicates is shown in parentheses. ^†^MD/SCPK, M Dalod, S Chan, P Kastner; AYR, AY Rudensky; SB, S Bondada; BP, B Pulendran; SA, S Akira; RM, R Medzhitov; CK, C Kim; MH, M Hikida; CB/DM, C Benoist, D Mathis; FT, F Takei; CRES, C Reis e Sousa; FH, F Housseau; FRS, FR Sharp; CAKB, CAK Borrebaeck; SYH, S Yla-Herttuala; LZH, L Ziegler-Heitbrock; MVD, MV Dhodapkar. ^‡^Shown in the indicated figure in this study. BM-DC, mouse bone-marrow derived GM-CSF DCs; BM-MΦ, mouse bone marrow-derived M-CSF macrophages; monocyte-derived MΦ, monocyte-derived M-CSF macrophages; NA, not applicable; PBMC-derived MΦ, human peripheral blood mononuclear cell-derived M-CSF macrophages; peritoneal MΦ, peritoneal mouse macrophages.

To verify the quality of the datasets mentioned above, we analyzed signal intensities for control genes whose expression profiles are well documented across the cell populations under consideration. Expression of signature markers were confirmed to be detected only in each corresponding population (see Table 2 for mouse data and Table 3 for human data). For example, *Cd3 *genes were detected primarily in T cells and often to a lower extent in NK cells; the mouse *Klrb1c *(*nk1.1*) gene or the human *KIR *genes in NK cells; *Cd19 *in B cells; the mouse *Siglech *and *Bst2 *genes or the human *LILRA4 *(*ILT7*) and *IL3RA *(*CD123*) genes in pDCs; and *Cd14 *in myeloid cells. As expected, many markers were expressed in more than a single cell population. For example, in the mouse, *Itgax *(*Cd11c*) was found expressed to high levels in NK cells and all DC subsets; *Itgam *(*Cd11b*) in myeloid cells, NK cells, and CD11b cDCs; *Ly6c *at the highest level in pDCs but also strongly in many other leukocyte populations; and *Cd8a *in pDCs and CD8α cDCs. However, the analysis of combinations of these markers confirmed the lack of detectable cross-contaminations between DC subsets: only pDCs expressed high levels of *Klra17 *(*Ly49q*) and *Ly6c *together, while *Cd8a*, *ly75 *(*Dec205*, *Cd205*), and *Tlr3 *were expressed together at high levels only in CD8α cDCs, and *Itgam *(*Cd11b*) with *Tlr1 *and high levels of *Itgax *(*Cd11c*) only in CD11b cDCs. Thus, each cell sample studied harbors the expected pattern of expression of control genes and our data will truly reflect the gene expression profile of each population analyzed, without any detectable cross-contamination.

**Table 2:** Expression of control genes in mouse cells

			Dendritic cells			Lymphocytes			
				
Probe set ID	Gene	Myeloid cells	pDC	CD8α DC	CD11b DC	NK	CD8 T	CD4 T	B
1419178_at	*Cd3g*	40 ± 10	<20	<20	<20	97 ± 31	2,074 ± 287	1,974 ± 478	22 ± 3
1422828_at	*Cd3d*	111 ± 14	<20	<20	<20	214 ± 16	2,815 ± 11	4,520 ± 1,414	21 ± 2
1422105_at	*Cd3e*	115 ± 30	27 ± 10	22 ± 2	23 ± 5	26 ± 9	387 ± 58	522 ± 210	26 ± 10
1426396_at	*Cd3z*	<20	<20	<20	<20	1,147 ± 81	1,545 ± 10	2,117 ± 482	25 ± 9
1426113_x_at	*Tcra*	83 ± 8	<20	23 ± 4	<20	116 ± 39	2,517 ± 42	5,601 ± 1,818	34 ± 13
1419696_at	*Cd4*	24 ± 2	1,233 ± 144	<20	369 ± 49	<20	<20	1,052 ± 73	<20
1450570_a_at	*Cd19*	190 ± 44	<20	<20	<20	<20	<20	23 ± 5	2,259 ± 292
1449570_at	*Klrb1c (NK1.1)*	<20	<20	<20	<20	2,328 ± 112	<20	25 ± 7	<20
1425436_x_at	*Klra3 (Ly49C)*	130 ± 11	24 ± 3	156 ± 0	242 ± 31	9,186 ± 479	170 ± 61	70 ± 42	<20
1450648_s_at	*H2-Ab1*	6,887 ± 84	7,339 ± 5	9,101 ± 100	9,056 ± 277	81 ± 6	83 ± 56	978 ± 11	7,028 ± 239
1419128_at	*Itgax (CD11c)*	454 ± 5	1,928 ± 169	2,827 ± 454	4,701 ± 56	3,403 ± 45	108 ± 44	22 ± 2	<20
1457786_at	*Siglech*	31 ± 4	3,454 ± 536	24 ± 5	<20	<20	<20	33 ± 13	<20
1425888_at	*Klra17 (Ly49Q)*	98 ± 4	3,413 ± 116	30 ± 14	163 ± 2	28 ± 11	24 ± 6	38 ± 10	<20
1424921_at	*Bst2 (120G8)*	2,364 ± 149	5,571 ± 718	237 ± 30	196 ± 44	61 ± 24	162 ± 12	90 ± 3	88 ± 32
1421571_a_at	*Ly6c*	4,420 ± 261	8,255 ± 151	98 ± 5	30 ± 8	2,082 ± 365	4,530 ± 229	1,789 ± 1,242	302 ± 303
1422010_at	*Tlr7*	439 ± 13	846 ± 40	<20	322 ± 45	<20	<20	22 ± 2	118 ± 83
1440811_x_at	*Cd8a*	<20	337 ± 134	825 ± 44	<20	<20	1,235 ± 227	22 ± 2	<20
1449328_at	*Ly75 (Dec205)*	249 ± 27	<20	159 ± 4	22 ± 3	24 ± 6	170 ± 29	79 ± 1	21 ± 1
1422782_s_at	*Tlr3*	27 ± 2	25 ± 3	3,376 ± 159	287 ± 14	<20	<20	<20	52 ± 45
1422046_at	*Itgam (CD11b)*	956 ± 57	<20	<20	162 ± 1	188 ± 38	<20	<20	21 ± 1
1449049_at	*Tlr1*	1,218 ± 54	31 ± 15	101 ± 4	1,601 ± 92	<20	889 ± 109	498 ± 103	1,141 ± 484
1417268_at	*Cd14*	7,649 ± 169	187 ± 52	107 ± 0	115 ± 34	<20	<20	31 ± 8	27 ± 12
1449498_at	*Marco*	174 ± 19	<20	<20	<20	<20	<20	<20	<20
1460282_at	*Trem1*	415 ± 19	<20	<20	<20	<20	<20	<20	<20

**Table 3:** Expression of control genes in human cells

		Lymphocytes				Dendritic cells			Myeloid cells	
				
Probe set ID	Genes	NK	CD8 T	CD4 T	B	pDC	BDCA1	BDCA3	Mono	Neu
206804_at	*CD3G*	858 ± 71	1,760 ± 241	1,975 ± 132	53 ± 6	<50	<50	<50	<50	52 ± 4
213539_at	*CD3D*	5,413 ± 238	7,134 ± 635	6,291 ± 285	276 ± 24	<50	<50	51 ± 2	112 ± 9	276 ± 4
205456_at	*CD3E*	247 ± 21	569 ± 67	679 ± 91	<50	<50	<50	<50	<50	<50
210031_at	*CD3Z*	8,688 ± 181	5,223 ± 218	4,749 ± 123	2,996 ± 217	56 ± 10	60 ± 17	54 ± 7	914 ± 96	132 ± 15
209671_x_at	*TCR@*	147 ± 16	3,127 ± 260	3,462 ± 170	71 ± 7	<50	<50	<50	<50	111 ± 16
205758_at	*CD8A*	911 ± 26	5,259 ± 217	67 ± 10	79 ± 16	<50	<50	<50	<50	99 ± 7
207979_s_at	*CD8B*	77 ± 9	3,596 ± 299	<50	<50	<50	<50	<50	<50	53 ± 5
203547_at	*CD4*	<50	<50	391 ± 20	83 ± 20	1,301 ± 119	1,004 ± 74	278 ± 61	205 ± 34	<50
206398_s_at	*CD19*	<50	51 ± 1	<50	1,726 ± 115	<50	<50	<50	57 ± 12	<50
212843_at	*NCAM1 (CD56)*	2,074 ± 96	144 ± 14	65 ± 2	135 ± 9	<50	<50	82 ± 17	52 ± 3	<50
207314_x_at	*KIR3DL2*	3,131 ± 172	454 ± 14	227 ± 18	265 ± 16	<50	<50	<50	59 ± 8	<50
208203_x_at	*KIR2DS5*	3,472 ± 140	444 ± 7	236 ± 10	284 ± 14	<50	<50	<50	<50	<50
239975_at	*HLA-DPB2*	<50	<50	<50	63 ± 22	777 ± 701	1,565 ± 519	2,056 ± 577	<50	<50
210184_at	*ITGAX (CD11c)*	1,017 ± 50	112 ± 37	166 ± 17	752 ± 45	74 ± 21	2,151 ± 430	729 ± 98	1,284 ± 115	2,133 ± 196
210313_at	*LILRA4 (ILT7)*	226 ± 10	117 ± 13	346 ± 42	1,109 ± 76	7,916 ± 612	230 ± 16	1,659 ± 1,183	524 ± 41	<50
206148_at	*IL3RA (CD123)*	84 ± 3	59 ± 8	91 ± 2	324 ± 9	4,728 ± 365	61 ± 10	116 ± 110	120 ± 3	74 ± 12
1552552_s_at	*CLEC4C (BDCA2)*	93 ± 6	61 ± 5	99 ± 4	408 ± 9	6,789 ± 737	76 ± 39	859 ± 434	217 ± 8	175 ± 25
205987_at	*CD1C (BDCA1)*	76 ± 8	61 ± 12	159 ± 8	1,715 ± 85	64 ± 23	8,313 ± 272	722 ± 845	560 ± 59	<50
204007_at	*FCGR3B (CD16)*	459 ± 54	115 ± 24	65 ± 5	322 ± 46	63 ± 23	<50	51 ± 1	160 ± 11	5,554 ± 57
201743_at	*CD14*	94 ± 3	139 ± 5	343 ± 5	1,274 ± 113	<50	202 ± 183	<50	7,638 ± 446	4,621 ± 374
205786_s_at	*ITGAM (CD11b)*	5,688 ± 116	1,980 ± 147	1,161 ± 71	2,513 ± 117	360 ± 184	703 ± 28	86 ± 63	5,541 ± 193	5,232 ± 576
208982_at	*PECAM1 (CD31)*	2,232 ± 48	2,144 ± 91	1,487 ± 58	4,644 ± 102	3,834 ± 601	2,825 ± 290	2,680 ± 363	5,479 ± 219	7,699 ± 853
205898_at	*CX3CR1*	10,056 ± 53	6,633 ± 232	4,351 ± 170	6,055 ± 263	262 ± 45	1,296 ± 84	362 ± 419	5,717 ± 451	616 ± 21
39402_at	*IL1B*	69 ± 6	72 ± 7	52 ± 3	209 ± 27	<50	195 ± 131	69 ± 27	198 ± 9	2,920 ± 183
202859_x_at	*IL8*	95 ± 7	77 ± 6	72 ± 5	385 ± 26	218 ± 185	90 ± 9	680 ± 561	310 ± 17	8,685 ± 776
207094_at	*IL8RA*	199 ± 30	74 ± 8	81 ± 12	82 ± 2	<50	61 ± 9	67 ± 1	90 ± 1	4,784 ± 521

Mono, monocyte; neu, neutrophil.

### LN-DCs constitute a specific leukocyte family that includes pDCs in both the human and the mouse

**Figure 1:**
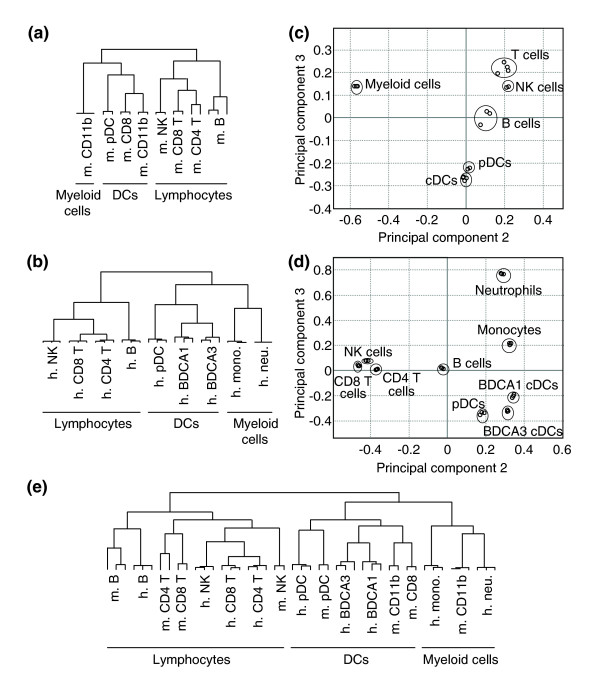
Clustering of mouse and human leukocyte subsets. Hierarchical clustering with complete linkage was performed on the indicated cell populations isolated from: **(a) **mouse, **(b) **human, and **(e) **mouse and human. PCA was performed on the indicated cell populations isolated from: **(c) **mouse and **(d) **human. Mono, monocytes; neu, neutrophils.

**Figure 2:**
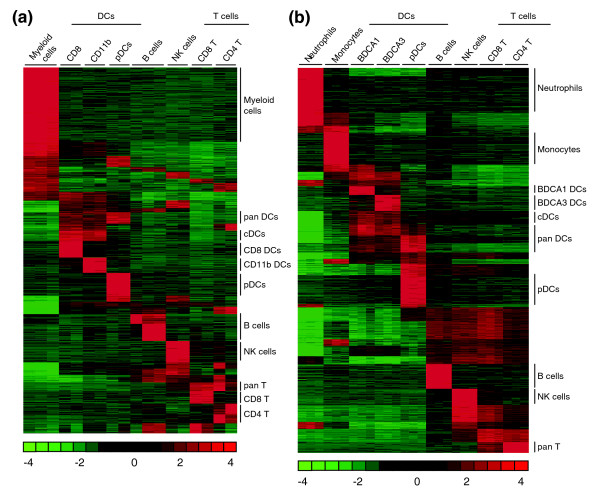
FCM partitional clustering. FCM partitional clustering was performed on the mouse and human gene chip datasets. **(a) **FCM partitional clustering for mouse data. **(b) **FCM partitional clustering for human data. The color scale for relative expression values as obtained after log_10 _transformation and median centering of the values across cell samples for each gene is given below the heat map.

To determine whether LN-DCs may constitute a specific leukocyte family, we first evaluated the overall proximity between LN-DC subsets as compared to lymphoid or myeloid cell types, based on the analysis of their global gene expression program. For this, we used hierarchical clustering with complete linkage [32], principal component analysis (PCA) [33], as well as fuzzy c-means (FCM) partitional clustering approaches [34]. Hierarchical clustering clearly showed that the three LN-DC subsets studied clustered together, both in mouse (7,298 genes analyzed; Figure 1a) and human (11,507 genes analyzed; Figure 1b), apart from lymphocytes and myeloid cells. The close relationship between all the DC subsets in each species was also revealed by PCA for mouse (Figure 1c) and human (Figure 1d). Finally, FCM clustering also allowed clear visualization of a large group of genes with high and specific expression levels in all DC subtypes (Figure 2, 'pan DC' clusters). These analyses, which are based on very different mathematical methods, thus highlight the unity of the LN-DC family. To investigate the existence of a core genetic program common to the LN-DC subsets and conserved in mammals, clustering of mouse and human data together was next performed. We identified 2,227 orthologous genes that showed significant variation of expression in both the mouse and human datasets. After normalization (as described in Materials and methods), the two datasets were pooled and a complete linkage clustering was performed. As shown in Figure 1e, the three major cell clusters, lymphocytes, LN-DCs, and myeloid cells, were obtained as observed above when clustering the mouse or human data alone. Thus, this analysis shows that DC subsets constitute a specific cell family distinct from the classic lymphoid and myeloid cells and that pDCs belong to this family in both mice and humans. All the LN-DC subsets studied therefore share a common and conserved genetic signature, which must determine their ontogenic and functional specificities as compared to other leukocytes, including other antigen-presenting cells.

### Identification and functional annotation of the conserved transcriptional signatures of mouse and human leukocyte subsets

Genes that are selectively expressed in a given subset of leukocytes in a conserved manner between mouse and human were identified and are presented in Table 4. Our data analysis is validated by the recovery of all the genes already known to contribute to the characteristic pathways of development or to the specific functions for the leukocyte subsets studied, as indicated in bold in Table 4. These include, for example, *Cd19 *and *Pax5 *for B cells [35], *Cd3e-g *and *Lat *for T cells [36], as well as *Ncr1 *[37] and *Tbx21 *(*T-bet*) [38] for NK cells. Similarly, all the main molecules involved in major histocompatibility (MHC) class II antigen processing and presentation are found selectively expressed in antigen-presenting cells (APCs). Indeed, a relatively high proportion of the genes selectively expressed in lymphocytes or in APCs has been known for a long time to be involved in the biology of these cells. However, we also found genes identified only recently as important in these cells, such as *March1 *[39] or *Unc93b1 *[40,41] for APCs, and *Edg8 *for NK cells [42]. Interestingly, we also identified genes that were not yet known to be involved in the biology of these cells, to the best of our knowledge, such as the E430004N04Rik expressed sequence tag in T cells, the *Klhl14 *gene in B cells, or the *Osbpl5 *gene in NK cells.

**Table 4:** Specific transcriptomic signatures identified in the leukocyte populations studied

	Expression ratio (log_2_) of specific genes*			
	
Cell type	3-4	2-3	1-2	0,4-1
Myeloid cells	*-*	*Steap4; Clec4d; Clec4e; Fpr1*	*Nfe2; Mpp1; Snca; Ccr1; Slc40a1; S100a9; ****Cd14***; ***Tlr4****; F5; Fcgr3; Fpr-rs2; ****Tlr2****; Abhd5; Gca; Atp6v1b2; Ier3; Sod2; Pilra; Slc11a1*	*Sepx1; Ninj1; Hp; Sdcbp; Bst1; Ifit1; S100a8; Adipor1; Bach1; Marcks; Pira2; Wdfy3; Ifrd1; Fcho2; ****Csf3r****; C5ar1; Cd93; Snap23; Cebpb; Clec7a; Yipf4; Hmgcr; Slc31a2; Fbxl5*
Pan-DC		***Flt3***	*Sh3tc1*	*Trit1; Bri3bp; Prkra; Etv6; Tmed3; Bahcc1; Scarb1*
cDC	*-*	*-*	*Arhgap22; Btbd4; Slamf8; 9130211I03Rik; Nav1*	***C2ta****; Avpi1; Spint1; Cs*
pDC		***Epha2****; Pacsin1; Zfp521; Sh3bgr*	*Tex2; Runx2; Atp13a2; Maged1; Tm7sf2; Tcf4; Gpm6b; Cybasc3*	*Nucb2; Alg2; Pcyox1; LOC637870; Scarb2; Dnajc7; Trp53i13; ****Plac8****; Pls3; ****Tlr7****; Ptprs; Bcl11a*
B cells	***Ebf1****; ****Cd19****; Klhl14*	***Bank1****; ****Pax5***	***Blr1****; Ralgps2; ****Cd79b****; ****Pou2af1****; Fcer2a; Cr2; ****Cd79a****; Fcrla*	*Ms4a1; ****Blk****; ****Cd72*** *; Syvn1; BC065085; Fcrl1; Phtf2; Tmed8; Grap; Pip5k3; * ***Pou2f2***
NK cells	*-*	***Ncr1***	***Tbx21****; Osbpl5*	*Rgs3; 1700025G04Rik; Plekhf1; ****Fasl****; Zfpm1; ****Edg8****; ****Cd160****; ****Klrd1****; ****Il2rb****; ****Il18rap****; Ctsw; ****Ifng****; ****Prf1****; Sh2d2a; Llgl2; Gpr178; Prkx; Gab3; ****Nkg7****; Cst7; Sntb2; Runx3; Myo6; F2r; Vps37b; Dnajc1; Gfi1*
Pan-T cells	*-*	***Camk4****; E430004N04Rik; ****Trat1***	***Cxcr6****; Tnfrsf25; Ccdc64; ****Plcg1***	***Cd3e****; ****Cd5****; Lrig1; ****Cd3g****; Ubash3a; ****Cd6****; ****Lat****; Bcl11b; ****Tcf7****; ****Icos***
CD8 T cells	*-*	*-*	*-*	*Gzmk*
CD4 T cells	*-*	***Ctla4***	*-*	***Icos****; Tnfrsf25; ****Cd5****; ****Cd28****; Trat1*
Lymphocytes	*-*	*-*	*Ablim1; Lax1; D230007K08Rik; Rasgrp1; ****Bcl2***	*Spnb2; Cdc25b; ****Ets1****; Sh2d2a; Ppp3cc; Cnot6l*
Myeloid, B, DC	*-*	***H2-DMb2****; ****H2-DMb1***	***C2ta****; ****March1****; Aldh2; Bcl11a; Btk*	***Ctsh****; ****H2-Eb1****; ****Cd74****; ****Ctsz****; Clic4; ****Kynu****; 5031439G07Rik; Nfkbie; ****Unc93b1***
Non-DC	***Gimap4***	*-*	*Vps37b*	***Lck****; Pde3b*

*Ratio expressed as Minimum expression among the cell types selected/Maximum expression among all other cell types. Genes already known to be preferentially expressed in the cell types selected are shown in boldface.

In contrast to the high proportion of documented genes selectively expressed in the cell types mentioned above, most of the genes specifically expressed in LN-DCs have not been previously associated with these cells and many have unknown functions. Noticeable exceptions are *Flt3*, which has been recently shown to drive the differentiation of all mouse [43-45] and human [46] LN-DC subsets [47], and *Ciita *(*C2ta*), which is known to specifically regulate the transcription of MHC class II molecules in cDCs [48]. Interestingly, mouse or human LN-DCs were found to lack expression of several transcripts present in all the other leukocytes studied here, including members of the *gimap *family, especially *gimap4*, which have been very recently shown to be expressed to high levels in T cells and to regulate their development and survival [49-51].

Thus, the identity of the gene signatures specific for the various leukocyte subsets studied highlights the sharp contrast between our advanced understanding of the molecular bases that govern the biology of lymphocytes or the function of antigen presentation and our overall ignorance of the genetic programs that specifically regulate DC biology. This contrast is enforced upon annotation of each of the gene signatures found with Gene Ontology terms for biological processes, molecular functions, or cellular components, and with pathways, or with interprotein domain names, using DAVID bioinformatics tools [52,53] (Table 5). Indeed, many significant annotations pertaining directly to the specific function of myeloid cells, lymphocyte subsets or APCs are recovered, as indicated in bold in Table 5. In contrast, only very few significant annotations are found for LN-DCs, most of which may not appear to yield informative knowledge regarding the specific functions of these cells.

**Table 5:** Selected annotations for the conserved transcriptomic signatures identified for the cell types studied

Cell type*	Annotation	Genes
Myeloid cells	**Defense response/response to pest, pathogen or parasite/inflammatory response**	*C5ar1, Sod2, Fcgr3, Tlr2, Ccr1, Ifrd1, Csf3r, Clec7a, Bst1, Ifit1, Clec4e, Tlr4, Clec4d, Cd14, Cebpb, Hp*
	**Response to bacteria or fungi/pattern recognition receptor activity/C-type lectin**	*SLC11A1, TLR2, TLR4, CLEC7A, Clec4e, Clec4d*
	**H_tollpathway: Toll-like receptor pathway**	*CD14, TLR2, TLR4*
	**Regulation of cytokine biosynthesis/positive regulation of TNF-α or IL-6 biosynthesis**	*Fcgr3, Tlr2, Tlr4, Cebpb, Clec7a*
	**Macrophage activation/mast cell activation/neutrophil chemotaxis**	*CD93, TLR4, Fcgr3, Csf3r*
Pan-DC	Binding	*ETV6, PRKRA, FLT3, SCARB1, TRIT1, BAHCC1, SH3TC1*
cDC	Nucleobase, nucleoside, nucleotide and nucleic acid metabolism	*NAV1, BTBD4, CIITA, SNFT*
	Molecular function unknown	*Btbd4, Avpi1, Arhgap22*
pDC	Transcription cofactor activity	*Maged1, Bcl11a, Tcf4*
	Integral to membrane	*TLR7, EPHA2, TMEPAI, SCARB2, ATP13A2, ALG2, CYBASC3, TM7SF2, GPM6B, PTPRS*
	Cellular component unknown	*Maged1, Sh3bgr, Cybasc3, Alg2, Plac8*
B cells	**MMU04662: B cell receptor signaling pathway/B cell activation**	*Cr2, Cd79a, Cd79b, Cd72, Cd19, Blr1, Ms4a1*
	**MMU04640: hematopoietic cell lineage**	*Cr2, Fcer2a, Ms4a1, Cd19*
	**Defense response/response to pest, pathogen or parasite/humoral immune response**	*PAX5, POU2F2, CR2, MS4A1, CD72, CD19, POU2AF1, BLR1, CD79A, CD79B, FCER2*
NK cells	**MMU04650: natural killer cell mediated cytotoxicity/apotosis**	*Klrd1, Ifng, Ncr1, Fasl, Prf1, Prf1, Plekhf1*
	**Defense response**	*IL18RAP, CTSW, IFNG, FASLG, CD160, NCR1, PRF1, KLRD1, CST7*
Pan-T cells	**HSA04660: T cell receptor signaling pathway/immunological synapse**	*CD3E, ICOS, PLCG1, LAT, CD3G, Trat1*
	**Defense response/immune response**	*Cd5, Icos, Cd3e, Ubash3a, Lat, Trat1, Cd3g*
	**HSA04640: hematopoietic cell lineage**	*CD3E, CD3G, CD5*
CD8 T cells	No annotations	*-*
CD4 T cells	**Defense response/immune response**	*Cd28, Icos, Cd5, Ctla4, Trat1*
	**M_ctla4pathway: the co-stimulatory signal during T-cell activation**	*Cd28, Icos, Ctla4*
Lymphocytes	**Immune response**	*BCL2, LAX1, ETS1*
Myeloid, B, DC	**Antigen presentation, exogenous antigen via MHC class II**	*H2-Eb1, H2-DMb2, H2-DMb1, Cd74*
	**HSA04612: antigen processing and presentation**	*HLA-DRB1, CIITA, CD74, HLA-DMB*
	**Defense response/immune response**	*H2-Eb1, H2-DMb2, H2-DMb1, Bcl11a, Cd74*
Non-DC	Phosphoric ester hydrolase activity	*LCK, PDE3B*

*The annotations recovered are written in boldface when they correspond to known specificities of the cell subset studied and are thus confirmatory of the type of analysis performed.

Thus, when taken together, our data show that LN-DC subsets constitute a specific family of leukocytes, sharing selective expression of several genes, most of which are still of unknown function. We believe that the identification of these genes selectively expressed in LN-DC subsets in a conserved manner between mouse and human will be very helpful for future investigation of the mechanisms regulating LN-DC biology by the generation and study of novel genetically manipulated animal models.

### Search for a genetic equivalence between mouse and human LN-DC subsets

**Figure 3:**
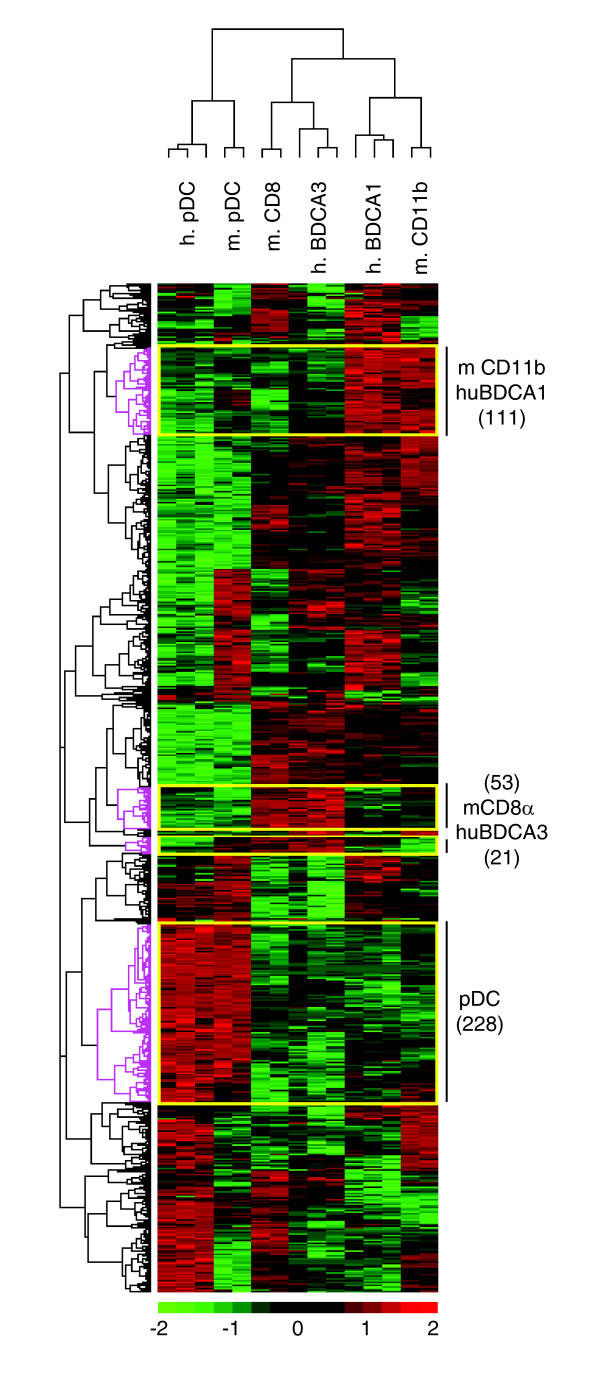
Conserved genetic signatures between mouse and human DC subsets. Hierarchical with complete linkage clustering was performed on the indicated DC populations isolated from mouse and human.

To search for equivalence between mouse and human LN-DC subsets, we examined their genetic relationships in the hierarchical clustering depicted in Figure 1e. Two observations can be made. First and remarkably, mouse and human pDCs clustered together. This result indicates a high conservation in their genetic program and establishes these two cell types as homologs. Indeed, human and mouse pDCs share a large and specific transcriptional signature (Table 4), with a number of genes comparable to those of the transcriptional signature of NK or T cells. To the best of our knowledge, most of these genes had not been reported to be selectively expressed in pDCs, with the exception of *Tlr7 *[31,54] and *Plac8 *(*C15*) [55]. Second, although mouse and human cDCs clustered together, the two cDC subsets of each species appeared closer to one another than to the subsets of the other species. Thus, no clear homology could be drawn between human and mouse cDC subsets in this analysis. However, it should be noted that known homologous human and mouse lymphoid cell types also failed to cluster together in this analysis and were closer to the other cell populations from the same species within the same leukocyte family. This is clearly illustrated for the T cell populations as mouse CD4 and CD8 T cells cluster together and not with their human CD4 or CD8 T cell counterparts (Figure 1e). Therefore, to further address the issue of the relationships between human and mouse cDC subsets, we used a second approach. We performed hierarchical clustering with complete linkage on the mouse and human LN-DC datasets alone (1,295 orthologous LN-DC genes), without taking into account the pattern of expression of each gene in the other leukocyte subsets as it may have hidden some degree of similarity between subsets clustering in the same branch. The results of the analysis of gene expression focused on DCs confirmed that mouse and human pDCs cluster together and apart from cDCs (Figure 3). Importantly, when analyzing the DC datasets alone, mouse CD8α and human BDCA3 cDCs on the one hand, and mouse CD11b and human BDCA1 cDCs on the other hand, clustered together and shared a conserved genetic signature (Figure 3 and Table 6). Thus, although a higher genetic distance is observed between mouse and human conventional DC subsets as opposed to pDCs, a partial functional equivalence is suggested between these cell types. The majority of the genes conserved between mouse CD8α and human BDCA3 cDCs versus mouse CD11b and human BDCA1 cDCs have unknown functions and have not been previously described to exhibit a conserved pattern of expression between these mouse and human cell types. Notable exceptions are *Tlr3 *[31,56] and the adhesion molecule Nectin-like protein 2 (*Cadm1*, also called *Igsf4*) [57], which have been previously described to be conserved between mouse CD8α and human BDCA3 cDCs. When comparing cDC to pDCs, a few genes already known to reflect certain functional specificities of these cells when compared to one another are identified. *Tlr7 *and *Irf7 *are found preferentially expressed in pDCs over cDCs, consistent with previous reports that have documented their implication in the exquisite ability of these cells to produce high levels of IFN-α/β in response to viruses [58-60]. *Ciita*, *H2-Ob*, *Cd83 *and *Cd86 *are found preferentially expressed in cDCs over pDCs, which is consistent with their higher efficiency for MHC class II antigen presentation and T cell priming [61].

**Table 6:** Conserved specific transcriptomic signatures of DC subsets compared to one another

	Expression ratio (log_2_) of specific genes*				
	
Cell type	>4	3-4	2-3	1-2	0,4-1
pDC	*Pacsin1; Sla2; 2210020M01Rik*	*-*	*Epha2; Sh3bgr; Ets1; Cobll1; Blnk; Myb; Sit1; Zfp521; Nucb2; Igj; Stambpl1; Ptprcap; Spib; Glcci1; Syne2; Ahi1; Atp13a2; Tcf4; Lair1*	*Runx2; LOC637870; Hs3st1; Asph; L3mbtl3; Tex2; Nrp1; Npc1; Maged1; Tm7sf2; ****Igh-6****; Csf2rb2; Ccr2; Cdk5r1; Fcrla; Rnasel; Arid3a; Rassf8; Tgfbr3; ****Tlr7****; Trp53i11; Ltb4dh; Arhgap24; Creb3l2; Itpr2; Bcl11a; Usp11; Gpm6b; Snx9; Hivep1; ****Irf7****; Cnp1; Cybasc3; Pcyox1; Aacs*	*Ifnar2; Ugcg; Kmo; Tspan31; Xbp1; Alg2; Txndc5; Abca5; Carhsp1; Ptp4a3; Lypla3; Cxxc5; Sema4c; Vamp1; Klhl9; BC031353; Cybb; Scarb2; Card11; Cdkn2d; 4931406C07Rik; Gimap8; Plxdc1; Lman1; 4631426J05Rik; Tcta; Mgat5; Ern1; Atp8b2; Lrrc16; Cln5; Rexo2; Atp2a3; Tspyl4; Anks3; Slc23a2; Gata2; Trp53i13; Slc44a2; Tmem63a; Dnajc7; Rhoh; Daam1; Lancl1; Aff3; Chst12; Unc5cl; Rwdd2; Armcx3; Vps13a; Mcoln2; Tm7sf3; Stch; Glt8d1; Pscd4; Ormdl3; 1110028C15Rik; Snag1; Prkcbp1; Klhl6; Cbx4; Pcmtd1; Bet1; Ccs; Tceal8; Dpy19l3; Pcnx; LOC672274; Sec11l3; Ctsb; Slc38a1; Ostm1; Acad11; Zbtb20; 1110032A03Rik; Ralgps2; Dtx3; Pls3; Ptprs; Zdhhc8; Rdh11; Bcl7a; Tbc1d2b*
cDC	*-*	*9130211I03Rik; Hnrpll; Fgl2; Id2; Slamf8*	*Chn2; Ddef1; Havcr2; A530088I07Rik; Rab32; Adam8; 2610034B18Rik; Dusp2; Btbd4; Pak1; Bzrap1; Anpep; Apob48r; Aif1*	*Arrb1; ****H2-Ob****; Arhgap22; Aytl1; 2810417H13Rik; Pik3cb; Nav1; Acp2; Tnfaip2; Tspan33; Ralb; Marcks; Epb4.1l2; Rab31; Aim1; Cias1; ****Cd86****; Cdca7; Rin3; Hk2; Actn1; Snx8; Cd1d1; Cxcl9; Sestd1; Anxa1; Il15; Ahr; Myo1f; Avpi1; Pde8a; Stom; Spint1; Kit; 1100001H23Rik; Specc1; Bcl6; Tpi1; Kcnk6; Efhd2; Cxcl16; Ddb2; ****C2ta*** *; Tgif; Pfkfb3; Ptpn12; Pitpnm1; Rtn1; Maff; Sgk; BB220380; Tes; Elmo1; Tm6sf1; Mast2; Stx11; Dhrs3; * ***Tlr2***	*Il18; Vasp; Ppfibp2; Itfg3; Wdfy3; Atad2; Hck; Cnn2; BC039210; Lima1; Fhod1; Klhl5; Flna; Egr1; Mrps27; Gas2l3; Atp2b1; Gypc; Lst1; 8430427H17Rik; Lmnb1; Junb; Irf2; Soat1; * ***Cd83****; Spg21; Nab2; Rbpsuh; Tiam1; Spfh1; Gemin6; Entpd1; Lzp-s; Lyzs; Slc8a1; Dusp16; Plscr1; Ptcd2; Slc19a2; Mthfd1l; Copg2; Dym; Limd2; Bag3; Csrp1; Ppa1; Nr4a2; Snx10; Hmgb3; Plekhq1; Oat; Rgs12; Numb; Hars2; Pacs1; Gtdc1; Ezh2; Swap70; Rasgrp4; Asahl; Susd3; Lrrk2; Sec14l1; Asb2; Txnrd2; E330036I19Rik; Sla; Fscn1; Nr4a1; Inpp1; Tdrd7; 4933406E20Rik; Usp6nl*
mCD8 and hBDCA3	*-*	*Clnk*	*Gcet2; BC028528; * ***Igsf4a***	*sept3; Sema4f; Fkbp1b; ****Tlr3****; Lima1; Dbn1; Plekha5; Fuca1; Fgd6; Snx22; Gfod1*	*Rasgrp3; Btla; Asahl; 4930506M07Rik; Lrrc1; 1700025G04Rik; Tspan33; Fnbp1; Itga6; Zbed3; 9030625A04Rik; Rab32; Ptcd2; Gas2l3; Rab11a; Ptplb; Cbr3; Pqlc2; Slamf8; St3gal5; 4930431B09Rik; Dock7; Stx3; Csrp1; Nbeal2; Gnpnat1; Slc9a9; Ncoa7*
mCD11b and hBDCA1	*-*	*-*	*Il1rn; Papss2; Pram1*	*Il1r2; Oas3; Rin2; Ptgs2; Csf1r; Tlr5; Centa1; Pygl; Igsf6; Csf3r; Tesc; Ncf2; S100a4; Rtn1; Cst7; Car2; Ifitm1; 1810033B17Rik; Lrp1; Dennd3; Ifitm3*	*Gbp2; Oas2; Ccl5; Pilra; Sirpa; Pla2g7; Ifitm2; Ms4a7; Cdcp1; Nfam1; BC013672; Slc7a7; Ripk2; Map3k3; Ripk5; Lactb; Rsad2; Parp14; D930015E06Rik; Gyk; Ank; Atp8b4; Emilin2; Arrdc2; Slc16a3; Fcgr3; Clec4a2; Ksr1; Itgax; Sqrdl; Hdac4; Rel; Pou2f2; Chka; Lyst; Ubxd5; Jak2; Cd300a; Lst1; Ssh1; Casp1; D12Ertd553e; Ogfrl1; Rin3; Cd302; Pira2*

*Ratio expressed as Minimum expression among the cell types selected/Maximum expression among all other cell types. Genes already known to be preferentially expressed in the cell types selected are shown in boldface.

The functional annotations associated with the genes selectively expressed in specific DC subsets when compared to the others are listed in Table 7. The most significant clusters of functional annotations in pDCs point to the specific expression in these cells of many genes expressed at the cell surface or in intracellular compartments, including the endoplasmic reticulum, the Golgi stack, and the lysosome. A cluster of genes involved in endocytosis/vesicle-mediated transport is also observed. This suggests that pDCs have developed an exquisitely complex set of molecules to sense, and interact with, their environment and to regulate the intracellular trafficking of endocytosed molecules, which may be consistent with the recent reports describing different intracellular localization and retention time of endocytosed CpG oligonucleotides in pDCs compared to cDCs [62,63]. The most significant clusters of functional annotations in cDCs concerns the response to pest, pathogens or parasites and the activation of lymphocytes, which include genes encoding TLR2, costimulatory molecules (CD83, CD86), proinflammatory cytokines (IL15, IL18), and chemokines (CXCL9, CXCL16), consistent with the specialization of cDCs in T cell priming and recruitment. Clusters of genes involved in inflammatory responses are found in both pDCs and cDCs. However, their precise analysis highlights the differences in the class of pathogens recognized, and in the nature of the cytokines produced, by these two cell types: IFN-α/β production in response to viruses by pDCs through mechanisms involving IRF7 and eventually TLR7; and recognition and killing of bacteria and production of IL15 or IL18 by cDCs through mechanisms eventually involving TLR2 or lysozymes. Many genes selectively expressed in cDCs are involved in cell organization and biogenesis, cell motility, or cytoskeleton/actin binding, consistent with the particular morphology of DCs linked to the development of a high membrane surface for sampling of their antigenic environment and for the establishment of interactions with lymphocytes. pDCs and cDCs also appear to express different arrays of genes involved in signal transduction/cell communication, transcription regulation and apotosis. A statistically significant association with lupus erythematosus highlights the proposed harmful role of pDCs in this autoimmune disease [64].

**Table 7:** Selected annotations for the conserved transcriptomic signatures identified for DC subsets when compared to one another

Cell type	Annotation	Genes
pDC	Endoplasmic reticulum	*Ern1, Lman1, Txndc5, Rdh11, Tm7sf2, Asph, Ormdl3, Stch, Nucb2, Ugcg, Itpr2, Bet1, Sec11l3, Atp2a3*
	Golgi stack	*BET1, HS3ST1, CHST12, SNAG1, LMAN1, MGAT5, GLCCI1, Pacsin1*
	Lysosome	*Lypla3, Npc1, Scarb2, Ctsb, Pcyox1, Cln5*
	Endocytosis/vesicle-mediated transport	*Bet1; Gata2; Igh-6; Lman1; Npc1; Pacsin1; Vamp1*
	Integral to plasma membrane	*EPHA2, SCARB2, CSF2RB, SIT1, ATP2A3, IFNAR2, VAMP1, PTPRS, SLC23A2, PTPRCAP, LANCL1, TM7SF2, CCR2, TSPAN31*
	Inflammatory response	*TLR7, CYBB, IRF7, CCR2, BLNK*
	Intracellular signaling cascade/I-κB kinase/NF-κB cascade	*SNAG1, SLC44A2, TMEPAI, CARD11, ERN1, SLA2, IFNAR2, CARHSP1, SNX9, RALGPS2, CXXC5, CCR2, BLNK, RHOH*
	Regulation of transcription, DNA-dependent/DNA binding/transcription regulator activity/RNA polymerase II transcription factor activity/IPR004827: Basic-leucine zipper (bzip) transcription factor	*1110028C15Rik; Aff3; Anks3; Arid3a; Bcl11a; Carhsp1; Cbx4; Cdkn2d; Creb3l2; Cxxc5; Ern1; Ets1; Gata2; Hivep1; Ifnar2; Irf7; Maged1; Myb; Nucb2; Prkcbp1; Runx2; Sla2; Spib; Tcf4; Tspyl4; Xbp1; Zbtb20*
	Systemic lupus erythematosus	*LMAN1, CCR2, ETS1*
	Regulation of apoptosis	*CDK5R1, CARD11, ERN1, CBX4, TXNDC5, CTSB*
cDC	Response to pest, pathogen or parasite/defense response/immune response/response to stress/inflammatory response/cytokine biosynthesis/response to bacteria/lymphocyte activation	*ANXA1; NR4A2; CIAS1; TLR2; CD83; CD86; IL18; CXCL16; MAST2; AIF1; CIITA; SNFT; Lzp-s, Lyzs; ENTPD1; CXCL9; PLSCR1; BCL6; SGK; TXNRD2; DDB2; AHR; IRF2; LST1; SOAT1; HLA-DOB; CD1D; IL15; Rbpsuh; Swap70; Hmgb3; Egr1*
	Cytoskeleton/actin binding/filopodium/cell motility	*FLNA; FHOD1; CNN2; MYO1F; ACTN1; VASP; EPB41L2; FSCN1; KLHL5; MARCKS; Epb4,1l2; Mast2; Aif1; Csrp1; Elmo1; LIMA1; LMNB1; STOM; Nav1, CXCL16, ANXA1*
	Morphogenesis/cell organization and biogenesis/neurogenesis	*Rasgrp4; Myo1f; Aif1; Pak1; Pacs1; Vasp; Tiam1; Lst1; Cnn2; Numb; Csrp1; Fhod1; Nav1; Rab32; Stx11; Ezh2; Epb4,1l2; Flna; Acp2; Elmo1; Ralb; Rab31; Id2; Tnfaip2; Txnrd2; Anpep; Il18; Rbpsuh, Nr4a2; Spint1*
	Signal transduction/cell communication/MMU04010:MAPK signaling pathway/regulation of MAPK activity/GTPase regulator activity/small GTPase mediated signal transduction/IPR003579:Ras small GTPase, Rab type	*ADAM8; AHR; ANXA1; ARRB1; Asb2; Avpi1; CD83; CD86; Chn2; CIAS1; CXCL9; Dusp16; DUSP2; Elmo1; ENTPD1; FLNA; Hck; IL15; IL18; INPP1; Kit; Lrrk2; Mast2; NR4A1; NR4A2; PAK1; PDE8A; PIK3CB; PPFIBP2; Rab31; Rab32; Ralb; Rasgrp4; RBPSUH; RGS12; Rin3; RTN1; Sla; SLC8A1; Snx10; Snx8; Tiam1; TLR2; Arhgap22; Ddef1; Rgs12; Usp6nl*
	Transcription regulator activity	*Junb, Id2, Asb2, Ddef1, Irf2, Nr4a2, C2ta, Nab2, Egr1, Nr4a1, Ahr, 9130211I03Rik, Tgif, Rbpsuh, Bcl6*
	Apoptosis	*Ahr, Nr4a1, Il18, Bag3, Cias1, Elmo1, Cd1d1, Sgk, Bcl6*
mCD8 and hBDCA3	Cell organization and biogenesis	*DBN1, RAB32, ITGA6, FGD6, RAB11A, SEMA4F*
	Intracellular signaling cascade/small GTPase mediated signal	*MIST, TLR3, SNX22; DOCK7; FGD6; RAB11A; RAB32; RASGRP3; sep3*
mCD11b and hBDCA1	Immune response/defense response/inflammatory response/positive regulation of cytokine production/response to pest, pathogen or parasite/antimicrobial humoral response/IPR006117:2-5-oligoadenylate synthetase	*IFITM3, PTGS2, POU2F2, LST1, GBP2, CCL5, OAS2, FCGR2A, NCF2, CSF1R, TLR5, CSF3R, IL1R2, CST7, IL1RN, NFAM1, IFITM2, IFITM1, LILRB2, OAS3, LYST, CLEC4A, IGSF6, HDAC4, PLA2G7, RIPK2, OAS2, OAS3; Rel; Fcgr3*
	Signal transduction/cell communication/signal transducer activity/positive regulation of I-κB kinase/NF-κB cascade/protein-tyrosine kinase activity/IPR003123:Vacuolar sorting protein 9; vesicle-mediated transport; endocytosis	*CASP1; CCL5; CD300A; CD302; CENTA1; CHKA; CLEC4A; CSF1R; CSF3R; FCGR2A; IFITM1; IGSF6; IL1R2; IL1RN; ITGAX; JAK2; KSR1; LILRB2; LRP1; LYST; MAP3K3; MS4A7; NFAM1; OGFRL1; REL; RIN2; RIN3; RIPK2; RIPK5; RTN1; TLR5; Fcgr3*
	Chemotaxis/cell adhesion	*ITGAX, CD300A, CSF3R, EMILIN2, CLEC4A, CCL5, Fcgr3*
	HSA04640:hematopoietic cell lineage	*CSF1R, CSF3R, IL1R2*
	Asthma. Atopy	*PLA2G7, CCL5,*

The mCD11b/hBDCA1 cDC cluster of genes comprises many genes involved in inflammatory responses and the positive regulation of the I-kappaB kinase/NF-kappaB cascade. A statistically significant association with asthma also highlights the proinflammatory potential of this cell type. Recently, it has been reported that the mouse CD11b cDC subset is specialized in MHC class II mediated antigen presentation *in vivo *[11]. In support of our findings here that mouse CD11b cDCs are equivalent to human BDCA1 cDCs, we found that many of the genes involved in the MHC class II antigen presentation pathway that were reported to be expressed to higher levels in mouse CD11b cDCs over CD8α cDCs [11] are also preferentially expressed in the human BDCA1 cDC subset over the BDCA3 one. These genes include five members of the cathepsin family (*Ctsb*, *Ctsd*, *Ctsh*, *Ctss*, and *Ctsw*) as well as *Ifi30 *and *Lamp1 *and *Lamp2 *(see Additional data file 2 for expression values). Thus, it is possible that, like the mouse CD11b cDC subset, human BDCA1 cDCs serve as a subset of DCs that are specialized in presenting antigen via MHC class II molecules. It is also noteworthy that mCD11b and hBDCA1 cDCs express high constitutive levels of genes that are known to be induced by IFN-α/β and that can contribute to cellular antiviral defense (*Oas2*, *Oas3*, *Ifitm1*, *Ifitm2*, *Ifitm3*).

No significant informative functional annotations are found for the mCD8α/hBDCA3 cDC gene cluster. However, groups of genes involved in cell organization and biogenesis or in small GTPase regulator activity are found and the study of these genes may increase our understanding of the specific functions of these cells. Mouse CD8α cDCs have been proposed to be specialized for a default tolerogenic function but to be endowed with the unique ability to cross-present antigen for the activation of naïve CD8 T cells within the context of viral infection [65]. It will be important to determine whether this is also the case for hBDCA3 cDCs. From this point of view, it is noteworthy that hBDCA3 cDCs selectively express *TLR3*, lack *TLR7 *and *TLR9*, and exhibit the highest ratio of *IRF8 *(*ICSBP*)/*TYROBP *(*DAP12*) expression, all of which have been shown to participate in the regulation of the balance between tolerance and cross-presentation by mouse CD8α cDCs [65,66].

### Use of leukocyte gene expression compendia to classify cell types of ambiguous phenotype or function

#### Interferon-producing killer dendritic cells

**Figure 4:**
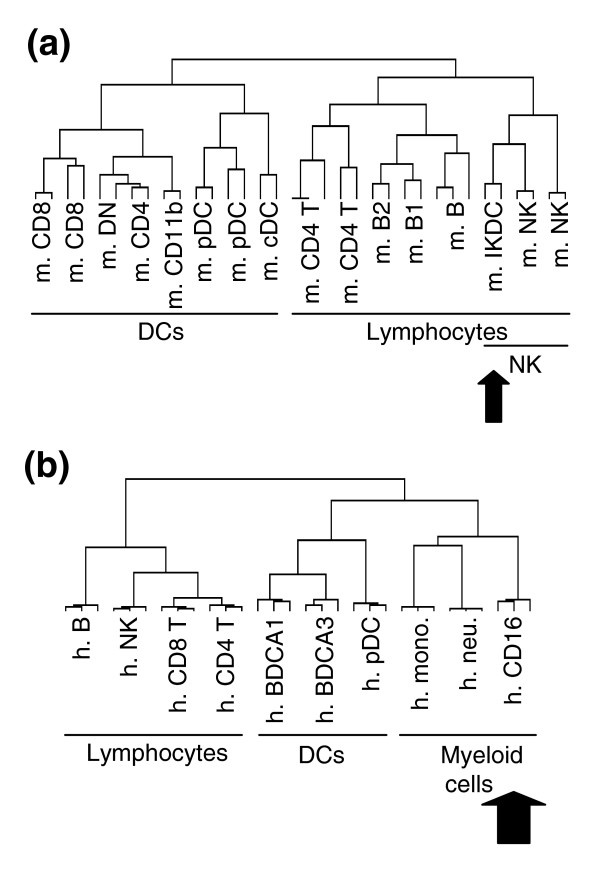
Clustering of mouse IKDCs and human CD16 cells. Hierarchical clustering with complete linkage was performed on the indicated cell populations isolated from: **(a) **mouse and **(b) **human. Mono, monocytes; neu, neutrophils.

A novel cell type has been recently reported in the mouse that presents mixed phenotypic and functional characteristics of pDCs and NK cells, IKDCs [15,16]. A strong genetic relationship between IKDCs and other DC populations was suggested. However, this analysis was based solely on comparison of the transcriptional profile of IKDCs to DCs and not to other cell populations [15]. As IKDCs were also reported to be endowed with antigen presentation capabilities [15] and to be present in mice deficient for the expression of RAG2 and the common γ chain of the cytokine receptors [16], they have been proposed to belong to the DC family rather than to be a subset of NK cells in a particular state of differentiation or activation. However, IKDCs have been reported to express many mRNA specific for NK cells and many of their phenotypic characteristics that were claimed to discriminate IKDCs from NK cells [16] are in fact consistent with classical NK cell features as recently reviewed [67], including the expression of B220 [68] and CD11c [69,70] (BD/Pharmingen technical datasheet of the CD11c antibody) [71]. To clarify the genetic nature of IKDCs, we reanalyzed the published gene chip data on the comparison of these cells with other DC subsets [15], together with available datasets on other leukocyte populations. We thus assembled published data generated on the same type of microarrays (Affymetrix U74Av2 chips) to build a second mouse compendium, allowing us to compare the transcriptomic profile published for the IKDCs (n = 2) with that of pDCs (n = 2), cDCs (n = 2) [15], CD8α^+^ (n = 2), CD4^+^ (n = 2) or double-negative (n = 2) cDC subsets [56], NK cells [72], CD4 T cells (n = 2), and B1 (n = 2) and B2 (n = 2) cells [18]. Information regarding the original sources and the public accessibility of the corresponding datasets are given in Table 1. As depicted in Figure 4a, the hierarchical clustering with complete linkage results of these data sets, together with our novel 430 2.0 data, clearly show that IKDCs cluster with NK cells, close to other lymphocytes, and not with DCs. Indeed, IKDCs express the conserved genetic signature of NK cells but not of DCs (Table 8 and Additional data file 4). Thus, these results strongly support the hypothesis that the cells described as IKDCs feature a specific subset of mouse NK cells that are in a particular differentiation or activation status, rather than a new DC subset.

**Table 8:** Expression of APC, DC and NK signature genes in IKDCs

									Ratio		
									
Probe set ID	Gene	CD8 DC	DN DC	CD4 DC	pDC	cDC	IKDC	NK	IKDC/DC	NK/DC	IKDC/NK
APC signature genes											
98035_g_at	*H2-DMb1*	2,701*	3,416	4,281	1,105	2,722	179	36	0.2	<0.1	5
92668_at	*Btk*	454	259	331	252	277	91	20	0.4	<0.1	5
94834_at	*Ctsh*	1,606	2,650	2,862	2,993	1,653	129	20	0.1	<0.1	6
94285_at	*H2-Eb1*	8,183	7,761	7,201	5,285	14,120	1,018	74	0.2	<0.1	14
101054_at	*Cd74*	9,094	7,810	7,313	5,158	12,258	1,031	55	0.2	<0.1	19
92633_at	*Ctsz*	520	1,246	1,171	887	750	117	44	0.2	<0.1	3
94256_at	*Clic4*	1,668	1,067	1,234	739	717	440	295	0.6	0.4	1
160781_r_at	*Unc93b1*	683	710	789	301	138	36	22	0.3	0.2	2
											
Pan-DC signature genes											
95295_s_at	*Flt3*	2,769	2,004	2,231	2,069	2,547	270	45	0.1	<0.1	6
100095_at	*Scarb1*	716	405	333	297	398	125	73	0.4	0.2	2
											
Non-DC signature genes											
96172_at	*Gimap4*	29	62	20	314	319	5,274	982	263	49	5
92398_at	*Vps37b*	111	139	44	76	56	462	159	11	4	3
161265_f_at	*Lck*	99	80	105	235	199	1,991	366	25	5	5
											
NK signature genes											
97781_at	*Ncr1*	20	20	20	73	39	1,483	120	20	2	12
97113_at	*Fasl*	20	28	20	22	30	440	263	15	9	2
102272_at	*Cd160*	75	107	62	82	58	780	246	7	2	3
100764_at	*Il2rb*	26	45	40	50	65	84	501	1	8	0.2
99334_at	*Ifng*	20	20	20	29	38	203	109	5	3	2
93931_at	*Prf1*	33	21	35	94	86	839	1,287	9	14	1
92398_at	*Vps37b*	111	139	44	76	56	462	159	11	4	3

#### Lineage^-^CD16^+^HLA-DR^+ ^cells

A subset of leukocytes characterized as lineage^-^CD16^+^HLA-DR^+ ^(hereafter referred to as CD16 cells) has been reported in human blood, and claimed to be a subpopulation of DCs based on their antigen-presentation capabilities. This subset segregates apart from BDCA1 and BDCA3 DCs and pDCs upon gene expression profiling [31]. It is not found in significant amounts in secondary lymphoid organs of healthy donors, contrary to pDCs and BDCA1 or BDCA3 cDCs. It expresses specific pattern recognition receptors, such as TLR4 and TLR8, and chemokine receptors, such as CX3CR1 and CMKOR1 [31], which were initially described to be preferentially expressed by monocytes in humans [73]. As the transcriptional relationship of CD16 cells with other known DC populations was originally established based solely on the transcriptional profile of DCs, we sought to better understand the nature of these cells. For this, we reanalyzed the global gene expression profile of CD16 cells in comparison to not only DC subsets but also to monocytes, neutrophils, and lymphocytes. The results depicted in Figure 4b clearly show that the CD16 cells cluster with neutrophils and monocytes and not with LN-DCs. Indeed, we find many genes that are expressed to much higher levels in monocytes or neutrophils and CD16 cells than in LN-DC subsets (Table 9 and Additional data file 2). Interestingly, *MAFB*, which has been described to inhibit the differentiation of DCs but to promote that of macrophages from hematopoeitic precursors [74], is expressed to much higher levels in CD16 cells and monocytes compared to DCs (average signal intensity of 6,263 in CD16 cells compared to 3,479 in monocytes, 65 in pDCs, 309 in BDCA1 DCs and <50 in BDCA3 DCs). CD16 cells also express to high levels many genes that are absent or only expressed to very low levels in LN-DCs compared to both lymphoid and myeloid cells, in particular many members of the *gimap *family. Reciprocally, many of the genes characterized above as specifically expressed in human and mouse LN-DCs are absent or expressed only to low levels in CD16 cells, in particular *FLT3 *and *SCARB1*. Thus, CD16 cells likely differentiate along the canonical myeloid lineage rather than belong to the LN-DC family. However, many genes are also specifically expressed to much higher levels in LN-DC subsets and CD16 cells than in monocytes, neutrophils and lymphocytes, attesting to the existence of biological functions common, and specific, to DC subsets and CD16 cells. Thus, these results strongly suggest that CD16 cells represent a particular subset of monocytes endowed with DC-like properties. One possibility is that CD16 cells are the naturally occurring equivalents of the 'monocyte-derived DCs' generated *in vitro*.

**Table 9:** Expression of APC, DC and myeloid signature genes in CD16 cells

		Dendritic cells			Myeloid cells			Ratio to DC		
					
Probe set ID	Gene	BDCA1	BDCA3	pDC	Mono	Neu	CD16 cells	CD16	Mono	Neu
APC signature genes										
203932_at	*HLA-DMB*	8,636*	7,929	5,894	5,194	173	2,581	0.3	0.6	<0.1
205101_at	*CIITA*	2,803	2,354	724	531	50	226	<0.1	0.2	<0.1
219574_at	*MARCH1*	587	777	544	1,214	58	810	1	2	<0.1
201425_at	*ALDH2*	9,279	7,841	6,034	8,504	706	1,760	0.2	0.9	<0.1
222891_s_at	*BCL11A*	569	747	4,502	310	50	213	<0.1	<0.1	<0.1
205504_at	*BTK*	1,120	822	1,132	1,409	281	1,786	2	1	0.3
202295_s_at	*CTSH*	6,197	2,528	1,211	3,949	75	2,440	0.39	0.6	<0.1
213831_at	*DQA1*	11,535	7,503	5,919	4,701	50	252	<0.1	0.4	<0.1
215536_at	*DQB2*	432	391	157	180	81	52	0.1	0.4	0.2
209312_x_at	*DRB1*	14,608	14,477	13,250	11,915	228	14,007	1	0.8	<0.1
209619_at	*CD74*	12,533	12,210	10,498	9,020	867	7,383	0.6	0.7	<0.1
210042_s_at	*CTSZ*	906	848	692	370	153	673	0.7	0.4	0.2
201560_at	*CLIC4*	920	305	663	3,023	165	354	0.4	3	0.2
217388_s_at	*KYNU*	2,414	1,059	2,204	3,516	50	3,738	2	1	<0.1
203927_at	*NFKBIE*	529	272	232	197	63	290	0.5	0.4	0.1
220998_s_at	*UNC93B1*	966	850	1,938	862	449	1,235	0.6	0.4	0.2
										
Pan-DC signature genes										
206674_at	*FLT3*	3,032	5,883	2,169	208	<50	<50	<0.1	<0.1	<0.1
219256_s_at	*SH3TC1*	1,263	899	1,128	392	166	858	0.7	0.3	0.1
218617_at	*TRIT1*	1,159	1,246	1,851	509	<50	339	0.2	0.3	<0.1
231810_at	*BRI3BP*	691	735	836	298	146	279	0.3	0.4	0.2
209139_s_at	*PRKRA*	846	1,067	1,440	316	74	497	0.3	0.2	<0.1
225764_at	*ETV6*	2,172	2,432	1,726	1,143	938	941	0.4	0.5	0.4
208837_at	*TMED3*	1,317	1,852	1,859	665	<50	1,022	0.6	0.4	<0.1
219218_at	*BAHCC1*	87	86	250	<50	<50	<50	0.2	0.2	0.2
1552256_a_at	*SCARB1*	325	425	942	165	128	59	<0.1	0.2	0.1
										
Non-DC signature genes										
219243_at	*GIMAP4*	68	<50	<50	4,404	3,504	1,334	20	65	52
221704_s_at	*VPS37B*	54	<50	<50	593	962	487	9	11	18
204891_s_at	*LCK*	<50	<50	<50	92	181	65	-	-	-
214582_at	*PDE3B*	78	<50	<50	129	625	114	1	2	8
										
Myeloid signature genes										
225987_at	*STEAP4*	<50	<50	<50	877	6,090	<50	-	-	-
1552773_at	*CLEC4D*	<50	<50	<50	452	520	<50	-	-	-
222934_s_at	*CLEC4E*	214	124	133	2,837	5,885	229	1	13	28
202974_at	*MPP1*	591	281	377	3,721	2,408	1,341	2	6	4
205098_at	*CCR1*	93	<50	115	3,712	3,627	106	1	32	31
223044_at	*SLC40A1*	769	276	321	5,018	3,444	<50	-	6	4
224341_x_at	*TLR4*	94	<50	<50	1,411	2,869	540	6	15	31
204714_s_at	*F5*	<50	<50	<50	1,392	2,313	<50	-	-	-
203561_at	*FCGR2A*	1,010	44	51	2,985	7,151	2,857	3	3	7
210772_at	*FPRL1*	<50	<50	<50	389	3,454	70	3	-	-
204924_at	*TLR2*	904	211	57	2,870	5,548	1,606	2	3	6
215223_s_at	*SOD2*	1,474	946	528	3,528	7,599	4,236	3	2	5
222218_s_at	*PILRA*	1,168	150	136	2,899	4,035	3,982	3	2	3
210423_s_at	*SLC11A1*	81	60	38	1,767	2,930	3,334	41	22	36
203045_at	*NINJ1*	357	66	71	1,104	3,129	1,934	5	3	9
201669_s_at	*MARCKS*	521	389	<50	2,449	3,224	1,730	3	5	6
207697_x_at	*LILRB2*	1,271	78	774	3,353	3,711	4,903	4	3	3
1553297_a_at	*CSF3R*	1,902	409	156	3,433	6,687	282	0.2	2	4
220088_at	*C5AR1*	56	34	93	2,316	5,099	3,824	41	25	55
221698_s_at	*CLEC7A*	3,229	4,295	79	6,642	7,061	5,680	1	2	2
204204_at	*SLC31A2*	442	187	<50	1,579	2,047	1,671	4	4	5

*Average expression across replicates. Mono, monocyte; neu, neutrophil.

#### *In vitro* GM-CSF derived DCs

**Figure 5:**
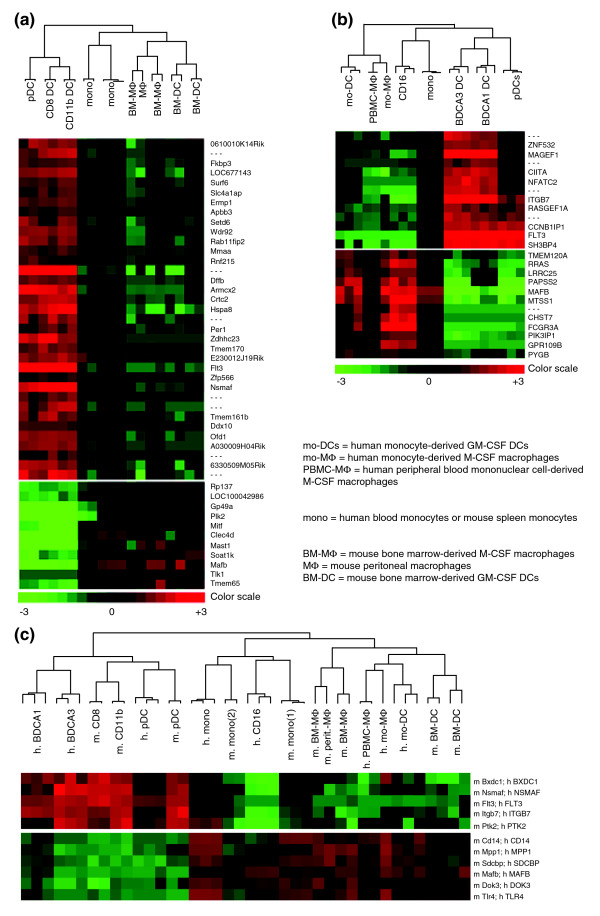
Clustering of *in vitro *GM-CSF derived DCs with monocytes, macrophages and LN-resident DCs. Hierarchical clustering with complete linkage was performed on the indicated cell populations isolated from: **(a) **mouse, **(b) **human, and **(c) **both. The heat maps used for illustration were selected as the two clusters of genes encompassing either *Flt3 *or *Mafb*, with a correlation cut-off for similarity of gene expression within each cluster at 0.8.

*In vitro* derived GM-CSF DCs are the most commonly used model to analyze DC biology. They are often used to investigate the interaction between DCs and other cell types or with pathogens, both in mouse (bone marrow (BM)-derived GM-CSF DCs) and human (monocyte-derived GM-CSF DCs). However, the relationship between these *in vitro *GM-CSF-derived DCs and the LN-DC subsets present *in vivo *in the steady state is not clear. A very recent publication suggests that *in vitro *derived GM-CSF mouse DCs may correspond to the DCs that differentiate from Ly6C^+ ^monocytes *in vivo *only under inflammatory conditions and appear specialized in the production of high levels of tumor necrosis factor-α and inducible nitric oxide synthase in response to intracellular bacteria, therefore differing from LN-DCs according to both ontogenic and functional criteria [75]. To gain further insights into the relationship between monocytes, macrophages, LN-DCs, and *in vitro *derived GM-CSF DCs, we thus compared their global gene expression profiling in both human and mouse, using publicly available gene chip data. Information regarding the original sources and the public accessibility of the corresponding datasets are given in Table 1. The results depicted in Figure 5 clearly show that the *in vitro *derived GM-CSF DCs cluster with monocytes and macrophages and not with the LN-DCs. This result was further confirmed by PCA, which also showed that both mouse and human GM-CSF DCs are close to macrophages, and distant from LN-DCs (Additional data file 6). Indeed, we found many genes that are expressed to much higher levels in monocytes, macrophages and *in vitro *derived GM-CSF DCs than in LN-DC subsets (Tables 10 and 11). As for human CD16 cells, these genes include the transcription factor *Mafb*. Reciprocally, some of the genes identified in this study as specific to LN-cDCs are expressed only to much lower levels in GM-CSF DCs. However and interestingly, compared to monocytes, *in vitro *derived GM-CSF DCs harbor stronger levels of other lymph node resident cDC-specific genes, including *scarb1*, *snft/9130211l03Rik*, *spint1*, *ctsh*, *C22ORF9/5031439G07Rik*, and *bri3bp*. Thus, *in vitro *derived GM-CSF DCs seem to harbor a strong myeloid gene signature but also express some of the LN-DC-specific genes, consistent with their myeloid ontogeny and their ability to exert myeloid-type functions but also with their acquisition of DC functional properties. In conclusion, our gene chip data analysis is consistent with a very recent report suggesting that *in vitro *derived GM-CSF mouse DCs correspond to inflammatory DCs and differ greatly from LN-DCs [75]. Indeed, several papers have recently established that *in vitro *derived FLT3-L DCs constitute the true equivalent of LN-DCs and constitute the only proper surrogate model currently available for their study [75-77].

**Table 10:** Comparison of the transcriptome of human GM-CSF monocyte-derived DCs to that of blood DCs

			Ratio to monocytes						
			
Probe set ID	Name	Mono	PBMC-MΦ	mo-MΦ	mo-DC	CD16	BDCA3	BDCA1	pDCs
Myeloid signature genes									
222934_s_at	CLEC4E	2,358	0.20	0.19	0.04	-	-	0.05	-
209930_s_at	**NFE2**	823	0.06	0.06	**0.89**	0.10	-	0.06	-
202974_at	**MPP1**	3,622	0.40	1.25	**0.68**	0.33	0.08	0.15	0.11
205098_at	**CCR1**	3,528	0.76	1.63	**1.83**	0.03	-	0.03	0.03
203535_at	S100A9	11,192	0.05	0.37	0.01	0.12	0.02	0.17	0.01
201743_at	**CD14**	8,096	0.44	1.13	**0.34**	0.01	-	0.02	0.01
224341_x_at	**TLR4**	1,417	0.13	1.10	**0.35**	0.34	-	0.06	-
203561_at	**FCGR2A**	2,946	0.18	0.80	**1.36**	0.85	-	0.33	0.02
204924_at	TLR2	3,220	0.14	0.80	0.32	0.54	0.08	0.31	0.02
218739_at	**ABHD5**	285	0.35	0.99	**0.67**	0.33	-	-	-
201089_at	**ATP6V1B2**	3,178	2.05	2.46	**1.70**	0.66	0.12	0.34	0.21
201631_s_at	**IER3**	2,042	0.42	1.74	**0.82**	0.10	0.06	0.14	0.12
222218_s_at	**PILRA**	2,709	0.73	1.24	**1.23**	1.25	0.05	0.39	0.05
210423_s_at	**SLC11A1**	1,713	0.47	0.82	**0.25**	1.75	0.04	0.05	-
203045_at	**NINJ1**	1,190	1.69	3.59	**3.41**	1.59	0.27	0.44	0.26
200958_s_at	**SDCBP**	11,323	0.87	1.16	**0.90**	0.61	0.33	0.40	0.26
202917_s_at	S100A8	15,661	0.02	0.41	0.01	0.11	0.01	0.27	0.03
217748_at	**ADIPOR1**	2,229	0.57	0.48	**1.16**	0.30	0.30	0.36	0.28
201669_s_at	**MARCKS**	2,340	0.84	2.57	**1.57**	0.65	0.16	0.20	-
207697_x_at	LILRB2	3,260	0.29	0.64	0.76	1.36	0.02	0.39	0.24
228220_at	**FCHO2**	619	4.50	4.04	**3.62**	0.76	0.35	0.26	0.23
1553297_a_at	CSF3R	3,121	0.42	0.69	0.37	0.08	0.11	0.52	0.04
220088_at	**C5AR1**	2,059	2.56	3.63	**1.30**	1.60	-	0.03	0.04
212501_at	**CEBPB**	3,490	3.26	3.23	**3.30**	1.26	0.06	0.49	0.06
221698_s_at	CLEC7A	6,596	0.24	0.55	0.63	0.74	0.62	0.46	0.01
209551_at	**YIPF4**	526	0.85	1.65	**1.91**	0.41	0.37	0.44	0.37
204204_at	**SLC31A2**	1,933	0.94	1.14	**0.69**	0.76	0.10	0.22	0.03
									
Pan-DC signature genes									
206674_at	**FLT3**	221	-	-	-	-	**24.01**	**12.76**	**9.26**
219256_s_at	**SH3TC1**	395	1.02	2.73	1.12	2.01	**2.22**	**3.01**	**2.86**
218617_at	**TRIT1**	498	0.49	0.58	0.86	0.71	**2.46**	**2.15**	**3.61**
231810_at	BRI3BP	301	0.98	1.42	1.99	0.98	2.35	2.10	2.70
209139_s_at	PRKRA	325	1.12	1.77	1.47	1.57	3.17	2.42	4.37
225764_at	ETV6	1,097	0.43	1.13	2.00	0.75	2.04	1.78	1.48
208837_at	TMED3	595	1.50	2.81	1.64	1.46	2.91	1.98	2.94
219218_at	BAHCC1	-	-	-	-	-	>1.7	>1.5	>4.7
1552256_a_at	SCARB1	151	8.98	6.58	7.21	-	2.33	1.70	5.30
									
cDC signature genes									
206298_at	ARHGAP22	-	>5.8	>6.5	>3.1	-	>6.2	>4.6	-
227329_at	BTBD4	-	>1.6	>2.8	>5.8	-	>9.3	>8.7	-
219386_s_at	SLAMF8	98	24.75	38.66	23.99	0.51	15.48	5.30	0.51
220358_at	SNFT	148	0.62	0.34	8.62	5.66	16.01	4.82	0.34
224772_at	**NAV1**	64	2.01	3.25	1.40	2.00	**23.87**	**10.50**	1.62
205101_at	**CIITA**	481	0.29	0.12	1.09	0.48	**4.51**	**5.28**	1.43
218631_at	AVPI1	-	>18.7	>31.3	>64.8	>1.6	>3.2	>7.0	-
202826_at	SPINT1	84	4.65	7.15	8.79	0.90	2.59	2.92	0.68
208660_at	CS	1,848	1.24	0.99	1.04	0.84	1.70	1.63	0.89
									
APC signature genes									
203932_at	HLA-DMB	5,137	1.28	0.64	1.37	0.44	1.45	1.62	1.14
219574_at	MARCH1	1,133	0.42	0.89	0.73	0.62	0.64	0.44	0.46
201425_at	**ALDH2**	8,782	0.51	0.54	0.34	0.18	**0.84**	**1.01**	**0.69**
222891_s_at	**BCL11A**	310	0.98	0.34	0.50	0.74	**2.40**	**1.73**	**14.23**
205504_at	BTK	1,372	0.29	0.47	0.64	1.13	0.58	0.75	0.81
202295_s_at	CTSH	3,755	1.76	2.37	2.09	0.56	0.63	1.57	0.31
209312_x_at	HLA-DRB1	12,737	1.02	0.57	1.34	1.11	1.12	1.11	1.00
209619_at	CD74	8,540	1.49	0.86	2.12	0.73	1.33	1.34	1.11
210042_s_at	CTSZ	369	0.76	1.13	17.00	1.66	2.13	2.17	1.83
201560_at	CLIC4	2,828	0.87	0.88	1.00	0.12	0.10	0.28	0.22
217388_s_at	KYNU	3,429	1.50	1.95	0.90	0.94	0.30	0.65	0.63
217118_s_at	C22orf9	1,617	3.33	3.46	2.77	1.43	1.85	1.79	1.04
203927_at	NFKBIE	173	3.30	9.96	3.13	1.45	1.39	2.60	1.25
220998_s_at	UNC93B1	847	0.60	1.31	0.97	1.31	0.99	1.06	2.27
									
Non-DC signature genes									
219243_at	**GIMAP4**	4,384	0.15	0.11	**0.19**	0.27	-	-	-
221704_s_at	**VPS37B**	559	0.26	0.90	**0.47**	0.80	-	-	-
204891_s_at	LCK	96	1.48	0.52	0.52	0.59	-	-	-
214582_at	**PDE3B**	144	2.82	2.99	**2.43**	0.76	-	0.51	-

^*^Average expression across replicates. Genes for which expression between monocyte-derived DCs and blood DCs or blood cDCs varies more than two-fold are shown in bold. mo-DC, monocyte-derived GM-CSF DC; mo-MΦ, monocyte-derived M-CSF macrophages; mono, monocyte; PBMC-MΦ, human peripheral blood mononuclear cell-derived M-CSF macrophages.

**Table 11:** Comparison of the transcriptome of mouse GM-CSF BM-derived DCs to that of spleen DCs

			Ratio to monocytes						
			
Probe set ID	Name	Mono	Mono(2)	MΦ	BM-MΦ	BM-DC	pDC	CD8 DC	CD11b DC
Myeloid signature genes									
1420804_s_at	**Clec4d**	4,934	0.65	0.49	0.75	**0.41**	-	-	-
1420330_at	**Clec4e**	5,511	0.11	0.22	0.23	**0.11**	-	-	-
1450808_at	**Fpr1**	119	1.91	-	5.55	**2.41**	-	-	-
1452001_at	**Nfe2**	139	1.44	-	-	**3.31**	-	-	-
1450919_at	**Mpp1**	1,888	0.15	2.05	1.75	**0.52**	0.23	0.09	0.07
1419609_at	**Ccr1**	403	1.27	4.04	0.53	**3.98**	0.2	-	-
1417061_at	**Slc40a1**	2,588	0.68	-	0.56	**0.07**	0.01	0.01	0.02
1448756_at	**S100a9**	8,664	1.2	-	0.01	**0.99**	0	0	-
1417268_at	**Cd14**	6,745	0.1	0.3	0.6	**0.19**	0.02	0.01	0.01
1418163_at	**Tlr4**	464	0.1	0.36	0.93	**0.66**	-	0.07	0.06
1448620_at	**Fcgr3**	1,471	2.02	3.56	2.15	**2.46**	-	0.02	0.07
1422953_at	**Fpr-rs2**	839	2.04	0.12	0.85	**1.58**	-	-	0.05
1419132_at	**Tlr2**	1,763	0.11	0.42	0.24	**0.48**	0.04	0.1	0.14
1417566_at	**Abhd5**	170	0.19	0.72	0.86	**2.2**	0.18	0.45	0.25
1415814_at	**Atp6v1b2**	1,556	0.22	2.75	1.57	**1.43**	0.18	0.27	0.24
1427327_at	**Pilra**	434	1.53	0.16	0.47	**2.29**	0.1	-	0.21
1418888_a_at	**Sepx1**	4,416	0.48	0.34	0.31	**0.56**	0.03	0.04	0.05
1438928_x_at	**Ninj1**	5,574	0.03	1.3	0.46	**0.36**	0.03	0.02	0.02
1448881_at	**Hp**	400	3.19	0.14	0.06	**3.09**	-	-	-
1449453_at	**Bst1**	340	1.08	4.97	0.58	**1.61**	0.21	0.51	-
1419394_s_at	**S100a8**	10,190	1.37	0.01	0.01	**0.66**	-	0	-
1437200_at	**Fcho2**	311	1.09	1.32	1.06	**0.76**	0.28	0.2	0.33
1418806_at	**Csf3r**	2,598	0.2	0.14	0.19	**0.11**	-	-	0.03
1439902_at	**C5ar1**	317	8.21	0.19	1.63	**0.37**	-	-	-
1456046_at	**Cd93**	1,559	0.1	0.49	1.18	**0.33**	0.02	-	-
1418901_at	**Cebpb**	3,797	0.14	0.7	0.22	**0.42**	0.02	0.01	0.02
1420699_at	**Clec7a**	2,748	0.83	2.62	0.44	**1.71**	0.08	0.06	0.54
									
Pan-DC signature genes									
1419538_at	**Flt3**	51	0.74	-	-	0.7	**16.2**	**25.32**	**17.78**
1427619_a_at	Sh3tc1	-	>1.1	>6.8	>2.8	>4.9	>5.2	>6.5	>4.6
1424489_a_at	**Trit1**	54	7.28	0.44	0.76	1.23	**9.03**	**11.53**	**8.63**
1428744_s_at	**Bri3bp**	161	0.84	0.6	1.44	3.28	**6.09**	**7.24**	**5.98**
1448923_at	Prkra	72	1.28	0.77	2.89	2.57	4.45	7.88	3.63
1434880_at	**Etv6**	140	5.39	1.52	0.74	1.75	**5.79**	**6.02**	**7.78**
1416108_a_at	Tmed3	154	0.81	3.74	2.63	4.65	10.17	4.48	3
1436633_at	**Bahcc1**	41	1.77	-	0.83	-	**1.8**	**3.88**	**2.35**
1437378_x_at	Scarb1	97	5.02	1.25	2.61	3.17	7.41	8.27	4.05
									
cDC signature genes									
1435108_at	**Arhgap22**	63	-	-	2.37	0.57	0.59	**10.65**	**4.43**
1429168_at	**Btbd4**	129	0.19	0.27	-	0.47	0.81	**3.89**	**3.8**
1425294_at	**Slamf8**	146	1.06	39.89	1.83	1.77	0.39	**8.48**	**5.27**
1453076_at	**9130211I03Rik**	36	1.61	2.85	1.03	13.11	0.62	**30.94**	**25.64**
1436907_at	**Nav1**	102	1.59	0.74	2.63	1.96	1.21	**6.08**	**13.14**
1421210_at	**C2ta**	125	0.17	1.79	0.19	0.93	1.46	**5.94**	**5.43**
1423122_at	**Avpi1**	150	0.32	-	0.2	0.86	0.61	**2.47**	**7.62**
1416627_at	Spint1	-	>1.5	>1.1	-	>22.9	>1.6	>25.7	>30.6
1450667_a_at	Cs	396	2.47	0.9	1.19	3.54	2.83	4.64	4.5
									
APC signature genes									
1419744_at	H2-DMb2	451	0.12	0.1	0.08	1.47	0.45	0.48	1.69
1443687_x_at	H2-DMb1	547	0.56	0.13	0.11	1.56	1.06	0.82	3.13
1434955_at	March1	80	32.64	0.83	1.51	3.48	3.73	13.4	8.57
1448143_at	Aldh2	867	0.47	2.14	2.07	1.32	0.95	0.65	0.45
1419406_a_at	**Bcl11a**	60	1.47	0.34	-	0.71	**20.41**	**7.63**	**9.19**
1422755_at	Btk	416	0.56	0.76	1.3	1.15	0.88	1.45	1.17
1418365_at	Ctsh	1,393	0.81	3.9	2.19	2.15	3.69	1.24	2.16
1417025_at	H2-Eb1	6,385	0.13	0.39	0.04	0.8	0.9	1.31	1.33
1425519_a_at	Cd74	8,377	0.36	0.95	0.2	0.9	0.83	0.97	0.98
1417868_a_at	Ctsz	7,061	0.05	1.16	0.95	0.85	0.5	0.3	0.49
1423393_at	Clic4	2,807	0.07	2.04	0.84	0.57	0.69	0.72	0.67
1430570_at	Kynu	31	1.23	-	-	3.21	12.87	5.16	11.56
1435745_at	5031439G07Rik	356	0.95	0.73	2.76	2.51	3.23	3.14	4.28
1458299_s_at	Nfkbie	767	0.4	0.62	0.1	0.44	1.25	0.65	1.27
1423768_at	Unc93b1	663	0.1	2.27	2.69	1.46	1.2	0.93	0.91
									
Non-DC signature genes									
1424375_s_at	Gimap4	362	0.14	0.29	-	0.1	0.11	-	0.11
1424380_at	Vps37b	313	0.44	0.46	0.45	0.26	0.28	0.28	0.27
1425396_a_at	Lck	118	-	0.57	0.2	0.32	0.21	-	0.17
1433694_at	Pde3b	352	0.69	0.15	0.16	0.42	-	0.65	0.35

^*^Average expression across replicates. Genes for which expression between mouse bone-marrow derived GM-CSF DCs (BM-DCs) and spleen DCs or spleen cDCs varies more than two-fold are shown in bold. BM-MΦ, mouse bone marrow-derived M-CSF macrophages; MΦ, peritoneal mouse macrophages; mono, mouse spleen monocytes from the SB laboratory; mono(2), mouse spleen monocytes from the BP laboratory, as listed in Table 1.

## Discussion

By performing meta-analyses of various datasets describing global gene expression of mouse spleen and human blood leukocyte subsets, we have been able to identify for the first time conserved genetic programs common to human and mouse LN-DC subsets. All the LN-DC subsets examined here are shown to share selective expression of several genes, while harboring only low levels of other transcripts present in all other leukocytes. These analyses indicate that LN-DCs, including pDCs, constitute a specific family of leukocytes, distinct from those of classic lymphoid or myeloid cells. Furthermore, we demonstrate a striking genetic proximity between mouse and human pDCs, which are shown for the first time to harbor a very distinct transcriptional signature as large and specific as that observed for NK cells or T cells. In contrast, a higher genetic distance is observed between mouse and human conventional DC subsets, although a partial functional equivalence is suggested between mCD8α and hBDCA3 cDCs on the one hand versus mCD11b and hBDCA1 cDCs on the other hand.

Our finding that LN-DCs constitute a distinct entity within immune cells raises the question of whether these cells form a distinct lineage in terms of ontogeny, or whether their shared gene expression profile (notably that between cDCs and pDCs) reflects a functional rather than a developmental similarity. To date, the place of both cDCs and pDCs in the hematopoietic tree is not clear [78,79]. A BM progenitor, named macrophage and dendritic cell progenitor (MDP), has been recently identified that specifically gives rise to monocytes/macrophages and to cDCs, but not to polymorphonuclear cells or to lymphoïd cells [80,81]. Under the experimental conditions used in the corresponding report, pDCs were not detected in the progeny of MDPs. Here, we show that the transcriptome programs of mouse spleen and human blood cDCs exhibit only a very limited overlap with that of monocytes/macrophages (Figure 2). This is consistent with the recent observation that monocytes can give rise to mucosal, but not splenic, cDCs, suggesting that splenic cDCs develop from MDPs without a monocytic intermediate [81]. While mouse pDCs have been argued to arise from both lymphoid or myeloid progenitors, their gene expression overlaps with lymphoid or myeloid cells are limited. Interestingly, a murine progenitor cell line that exhibits both cDC and pDC differentiation potential has been described recently [82], suggesting that putative pan-DC progenitors might also exist *in vivo*, which would be consistent with the gene profiling analyses presented here.

Our study identifies transcriptional signatures conserved between mouse and human, common to all LN-DC subsets examined, or specific to pDCs, cDCs, or individual cDC subsets. A genetic equivalence is suggested between mouse CD8α cDCs and human BDCA3 cDCs, and between mouse CD11b cDCs and human BDCA1 cDCs. In contrast to the genes selectively expressed in subsets of myeloid or lymphoid cells in a conserved manner between mouse and human, most of the genes specifically increased in all LN-DC subsets or in individual LN-DC subsets are currently uncharacterized. As a consequence, the functional annotations of the LN-DC transcriptional signatures appear much less informative than those for myeloid cells, lymphocytes or APCs. This highlights how much has already been deciphered regarding the molecular regulation of antigen presentation or lymphocyte biology, as opposed to how little we know about the genetic programs that determine the specific features of LN-DCs. We believe that our study provides a unique database resource for future investigation of the evolutionarily conserved molecular pathways governing specific aspects of the ontogeny and functions of leukocyte subsets, especially DCs.

It should be noted that many genes are found to be expressed to very high levels in specific subsets of either mouse or man while no orthologous gene has been identified in the other species. This could be due to a true absence of orthologous genes between these two vertebrate species, or to a lack of identification of an existing orthology relationship. It is also possible that some of the genes expressed only in mouse DCs or only in human DCs, and not conserved between the two species, might represent functional homologs, similar to what is observed for human KIR and mouse Ly49 NK cell receptors. This may be the case for the human LILRA4 (ILT7) and the mouse SIGLECH molecules, as both of them signal through immunoreceptor tyrosine-based activation motif (ITAM)-bearing adaptors to downmodulate IFN-α/β production by human and mouse pDCs, respectively, upon triggering of TLRs [83,84]. Thus, understanding the role in LN-DCs of genes identified here only in mouse or human might be important. The transcriptional signatures identified for mouse LN-DC subsets in this study have been confirmed by analyses of independent data recently published by others on mouse cDC subsets, B cells and T cells [11] or on cDCs and pDCs [15]. Most of the data for the mouse 430 2.0 compendium were generated in-house, with the exceptions being CD4 T cells and myeloid cells. In humans, we generated the data for non-DC populations, whereas data for DC subsets and CD16 cells were all generated by another group and retrieved from a public database. It is well known that datasets for the same cell type can vary considerably between laboratories. However, many of the genes identified as specific for each mouse LN-DC subset using our own data were confirmed by the analysis of other data independently generated by the groups of M Nussenzweig and R Steinman [11]. These data are given in Additional data file 5.

Our clustering analyses and PCA also showed relatively little dataset-dependent biases, and generally grouped related cell populations together, even if they were from different origins (see, for instance, the PCA clustering of *in vitro *derived GM-CSF DC samples, which originated from two independent datasets in Additional data file 6). In addition, we analyzed by real-time PCR the expression profile of 27 genes across mouse leukocyte subsets from biological samples independent of those used in the gene chips analysis. All the results were consistent with the gene chip data (Additional data file 7). We also confirmed specific expression of *PACSIN1 *in human pDCs at both the mRNA and protein levels (Additional data file 8). Finally, we believe that our approach validates the gene expression profile identified for leukocyte subsets in the strongest way possible, by demonstrating the evolutionary conservation between mouse and human. Indeed, the gene signatures that we describe here are based on genes found specifically expressed in putatively homologous subsets of mouse and human leukocytes compared to several other types of leukocytes. This approach does not rely solely on the use of independent biological samples of similar origin and on different techniques for measurement of the expression of mRNA. It actually shows that orthologous genes share the same specific expression pattern in putatively homologous immune cell subsets from two different species, under conditions where the markers used to purify the human and mouse cell populations, and the probes used to check the expression of the orthologous genes, differ considerably. Thus, we believe that the analyses presented here are extremely robust even though they were, in part, performed by creating compendia regrouping data generated by different laboratories for different cell types.

In addition to our discovery of transcriptional signatures specific to all LN-DCs or to LN-DC subsets, we demonstrate that, once identified, the transcriptional signatures of multiple cell types can be effectively used to help determine the nature of newly identified cell types of ambiguous phenotype or functions. In our attempt to appropriately place IKDCs and CD16 cells within the leukocyte family, we used the microarray data from the original reports aimed at characterizing these cells and compared them to the data from several other leukocyte populations. The conclusions of this analysis are in sharp contrast to those originally reported [15,31]. We believe that these opposing conclusions arise from the difference in the contextual framework within which our data and that of the previously mentioned studies were analyzed. Thus, the results of our analysis of the transcriptional signature of both IKDCs and CD16 cells emphasize the need to study the transcriptional signatures of individual cell populations in the context of multiple cell types of various phenotypes and functions. Finally, this approach also allowed us to confirm a very recent report that demonstrated that *in vitro *derived GM-CSF mouse DCs likely correspond to inflammatory DCs and greatly differ from LN-DCs, based on ontogenic and functional studies [75]. Thus, extrapolation to LN-DCs of the results of the cell biology and functional studies performed with *in vitro *derived GM-CSF DCs should only be made with extreme caution.

## Conclusion

This study comparing whole genome expression profiling of human and mouse leukocytes has identified for the first time conserved genetic programs common to all LN-DCs or specific to the plasmacytoid versus conventional subsets. In depth studies of these genetic signatures should provide novel insights on the developmental program and the specific functions of LN-DC subsets. The study in the mouse of the novel, cDC-specific genes identified here should accelerate the understanding of the mysteries of the biology of these cells in both mouse and human. This should help to more effectively translate fundamental immunological discoveries in the mouse to applied immunology research aimed at improving human health in multiple disease settings.

## Materials and methods

### Sorting of cell subsets

Duplicates of pDCs (Lin^-^CD11c^+^120G8^high^), CD8α cDCs (Lin^-^CD11c^high^CD8α^+^120G8^-/low^), CD11b cDCs (Lin^-^CD11c^high^CD11b^+^120G8^-/low^) and NK cells (NK1.1^+^TCRβ^-^) were sorted during two independent experiments from pooled spleens of untreated C57BL/6 mice. Splenic CD19^+ ^B lymphocytes, CD4 T cells and CD8 T cells were sorted in other independent experiments. Purity of sorted cell populations was over 98% as checked by flow cytometry (not shown).

### Processing of cell samples for the Affymetrix GeneChip assays

RNA was extracted from between 7.5 × 10^5 ^and 1.5 × 10^6 ^cells for each leukocyte subset with the Qiagen (Courtaboeuf, France) micro RNAeasy kit, yielding between 200 and 700 ng of total RNA for each sample. Quality and absence of genomic DNA contamination were assessed with a Bioanalyser (Agilent, Massy, France). RNA (100 ng) from each sample was used to synthesize probes, using two successive rounds of cRNA amplification with appropriate quality control to ensure full length synthesis according to standard Affymetrix protocols, and hybridized to mouse 430 2.0 chips (Affymetrix, Santa Clara, CA, USA). Raw data were transformed with the Mas5 algorithm, which yields a normalized expression value, and 'absent' and 'present' calls. Target intensity was set to 100 for all chips.

### Individual analysis of the mouse 430 2.0 or human U133 Plus 2.0 compendia

For each compendium, all datasets were normalized with the invariant rank method and only one representative dataset was kept for redundant ProbeSets targeting the same gene. The datasets were further filtered to eliminate genes with similar expression in all samples, by selecting only the genes expressed above 50 (respectively 100) in all the replicates of at least one population for the mouse (respectively human) datasets and whose expression across all samples harbored a coefficient of variation above the median of the coefficient of variation of all ProbeSets. The final dataset consisted of 7,298 (respectively 11,507) ProbeSets for the mouse 430 2.0 compendium (respectively human U133 Plus 2.0), representing individual genes with differential expression between *ex vivo *isolated cell subsets. The final dataset consisted of 12,857 (respectively 6,724) ProbeSets for the mouse 430 2.0 compendium (respectively human U133 Plus 2.0), representing individual genes with differential expression between LN-DCs, monocytes/macrophages and *in vitro *derived GM-CSF DCs. These datasets for *ex vivo *isolated cells are accessible as Excel workbooks in Additional data files 1 and 2. The software Cluster and Treeview were used to classify cell subsets according to the proximity of their gene expression pattern as assessed by hierarchical clustering with complete linkage.

We implemented a function in the Matlab software to perform PCA. This function computes the eigenvalues and eigenvectors of the dataset using the correlation matrix. The eigenvalues were then ordered from highest to lowest, indicating their relative contribution to the structure of the data. For both mouse and human datasets, the first principal component accounted for most of the information (54% and 68% for mouse and human, respectively) and was associated with a similar coordinate for all samples. This component thus reflected the common gene expression among the samples. Second and third components together represented 24% and 21%, respectively, of the information for mouse and human datasets, and thus accounted for a large part of the variability. The projection of each sample on the planes defined by these components was represented as a dot plot to generate the PCA figures.

Partitional clustering was performed using the FCM algorithm, which links each gene to all clusters via a vector of membership indexes, each comprised between 0 and 1 [34]. For both mouse and human datasets, we heuristically set the number of clusters to 30, and the fuzziness parameter m was taken as 1.2 (see [34] for the determination of m). Ten independent runs of the algorithm were performed, and the one minimizing the inertia criterion was selected [34]. A threshold value of 0.9 was taken to select probe sets most closely associated with a given cluster. This selection retained 4,062 and 4,751 probe sets from mouse and human datasets, respectively. Probe set clusters were then manually ordered to provide coherent pictures, which were visualized with Treeview.

### Meta-analysis of aggregated mouse and human datasets

We identified 2,227 orthologous genes that showed significant variation of expression in both the mouse 430 2.0 and U133 Plus 2.0 human datasets. This dataset is accessible as an Excel workbook in Additional data file 3. In order to compare the expression patterns of these genes between human and mouse, the log signal values for each of these genes were first normalized to a mean equal to zero and a variance equal to 1, independently in the mouse and human datasets, as previously described for comparing the gene expression program of human and mouse tumors [22,27]. The two normalized datasets were then pooled and a hierarchical clustering with complete linkage was performed. A similar analysis was performed for the comparison of human and mouse LN-DCs, monocytes, macrophages and *in vitro *derived GM-CSF DCs.

### Meta-analysis of mouse 430 2.0 and U74Av2 datasets

In order to classify the IKDCs based on the optimal gene signatures of the different cell subsets examined, with only minimal impact of differences in the experimental protocols used to prepare the cells and to perform the gene chips assays, the clustering of the cell populations was performed as a meta-analysis of our own mouse 430 2.0 dataset together with the published U74Av2 datasets. The Array Comparison support information of the NetAffyx™ analysis center (Affymetrix) was used to identify matched ProbSets between the two types of microarrays. Only one representative dataset was kept for redundant ProbeSets targeting the same gene. This yielded a set of 2,251 genes whose expression could be compared between the two datasets, using the same normalization method as described above. This dataset is accessible as Excel workbooks in Additional data file 4. As expected, this meta-analysis led to co-clustering of all the samples derived from identical cell types whether their gene expression had been measured by us on 430 2.0 microarrays or by others on U74Av2 microarrays, with the exception of the cDC population from [15], which segregated with pDCs rather than with the cDC subsets from the other datasets.

### Data mining

Gene lists were analyzed using the DAVID 'functional annotation chart' tool accessible on the NIAID website [52,53]. Different databases were used for these annotations: gene ontology (Amigo), knowledge pathways (KEGG), interactions (BIND), interprotein domains (INTERPRO), and disease (OMIM/OMIA). The annotations shown in Tables 5 and 7 were selected as the most highly significant terms retrieved by performing an over-representation study. To this end, a modified Fisher exact P value called the 'EASE score' was calculated to measure the enrichment in gene-annotation terms between the gene signature specific to the leukocyte subpopulation examined ('List') and the complete set of all the genes selected for the compendium analyzed ('Background'). The significance threshold was set at an EASE score below 0.05 in most instances, or below 0.1 for DC signatures that did not yield many highly significant terms as discussed in Results. Individual significant annotations encompassing many common genes or similar biological processes were regrouped using the 'Functional annotation clustering' tool of the DAVID software. More information on this type of analysis is available on the DAVID website [85].

### Public access to the raw data for the datasets analyzed in the paper

Our datasets for mouse DC subsets, NK cells, CD8 T cells, and B lymphocytes have been deposited in the Gene Expression Omnibus (GEO) database under reference number GSE9810. The references for download of the public data used from the original websites where they were first made available are given in Table 1. In addition, all raw transcriptomic data analyzed here have been regrouped on our website [86] and are available for public download.

## Abbreviations

APC, antigen-presenting cell; BM, bone marrow; cDC, conventional dendritic cell; CDP, common dendritic progenitor; DC, dendritic cell; FCM, fuzzy c-means; GEO, Gene Expression Omnibus; GM-CSF, granulocyte-macrophage colony stimulating factor; IFN, interferon; IKDC, interferon-producing killer dendritic cell; ITAM, immunoreceptor tyrosine-based activation motif; LN-DC, lymph node-resident DC; M-CSF, macrophage colony-stimulating factor; MDP, macrophage and dendritic cell progenitor; MHC, major histocompatibility; NK, natural killer; PCA, principal component analysis; pDC, plasmacytoid dendritic cell; TLR, toll-like receptor.

## Authors' contributions

SHR, TW, SC, PK, and MD designed the research; SHR, TW, CT, HX, MS, GB, AD and MD performed the research; EV and PP contributed new reagents/analytical tools; SHR, TW, CT, HX, DD, MS, FRS, SC, PK, and MD analyzed data; and SHR, TW, and MD wrote the paper.

## Note added in proof

During the review process of this paper, two reports were published in *Nature Immunology *that identified a common progenitor characterized as FLT3^+^M-CSF^+ ^for mouse LN-DCs (pDCs, CD8α cDCs and CD11b cDCs), devoid of any capability to generate lymphoid cells or monocytes/macrophages, and named common dendritic progenitor (CDP) [87,88]. This observation is thus consistent with our gene profiling analysis of human and mouse leukocytes. The question whether this pathway for LN-DCs is the major one, or just one possibility among others, including differentiation from monocytes, has been raised [89]. Our gene profiling data would suggest that most mouse LN-DCs derive from the recently identified CDP or MDP *in vivo*, without a monocytic intermediate, consistent with a recent report [81]. It also implies that a similar pathway must exist in humans. The relationship between the CDP and the MDP still remains to be established. Three reports have been published very recently in the *Journal of Experimental Medicine *that showed that IKDCs are a specific subset of NK cells, based on functional and ontogenic approaches comparing these cells to DCs and NK cells [90-92]. This is consistent with the results of our clustering analysis of IKDCs with other leukocyte subsets. Finally, two recent reports have identified a new transduction pathway in human pDCs involving a B cell receptor-like ITAM-signaling pathway [93,94]. This pathway involves the BLNK transduction molecule, which we have identified here as expressed to very high levels in mouse and human pDCs compared to the other LN-DCs (Table 6) and many other leukocytes. We believe that the conserved transcriptional signatures identified here for mouse and human LN-DC subsets will lead to many more discoveries for the understanding of the specialized functions of these cells.

## Additional data files

The following additional data are available. Additional data file 1 is a Microsoft Excel workbook with raw data for the mouse gene chip compendium. Additional data file 2 is a Microsoft Excel workbook with raw data for the human gene chip compendium. Additional data file 3 is a Microsoft Excel workbook with raw data for the human/mouse gene chip compendium. Additional data file 4 is a Microsoft Excel workbook with raw data for the IKDC gene chip compendium. Additional data file 5 is a Microsoft Excel workbook giving the mouse DC subset gene signatures according to our datasets with confirmation from two other independent datasets (one for pDCs and one for cDC subsets). Additional data file 6 is a figure showing the results of PCA for investigation of the relationships between *in vitro *derived GM-CSF DCs and LN-DCs in mouse and human. Additional data file 7 is a table giving real-time PCR data for the pattern of expression of 27 genes across mouse leukocyte subsets. Additional data file 8 is a figure illustrating PACSIN1 expression in human pDCs versus PBMCs by RT-PCR and western blotting.

## Supplementary Material

Additional file 1 Raw data for the mouse gene chip compendium. Click here for file

Additional file 2 Raw data for the human gene chip compendium. Click here for file

Additional file 3 Raw data for the human/mouse gene chip compendium. Click here for file

Additional file 4 Raw data for the IKDC gene chip compendium. Click here for file

Additional file 5 Mouse DC subset gene signatures according to our datasets with confirmation from two other independent datasets (one for pDCs and one for cDC subsets). Click here for file

Additional file 6 Results of PCA for investigation of the relationships between *in vitro *derived GM-CSF DCs and LN-DCs in mouse and human. Click here for file

Additional file 7 Real-time PCR data for the pattern of expression of 27 genes across mouse leukocyte subsets. Click here for file

Additional file 8 PACSIN1 expression in human pDCs versus PBMCs by RT-PCR and western blotting. Click here for file
