# Supplementary material for: Novel insights into the relationships between dendritic cell subsets in human and mouse revealed by genome-wide expression profiling
Source: Genome Biol. 2008 Jan 24;9(1):R17. doi: 10.1186/gb-2008-9-1-r17 (PMC2395256; doi:10.1186/gb-2008-9-1-r17)
Supplement: Additional file 6 — Results of PCA for investigation of the relationships between in vitro derived GM-CSF DCs and LN-DCs in mouse and human. [file gb-2008-9-1-r17-S6.pdf]

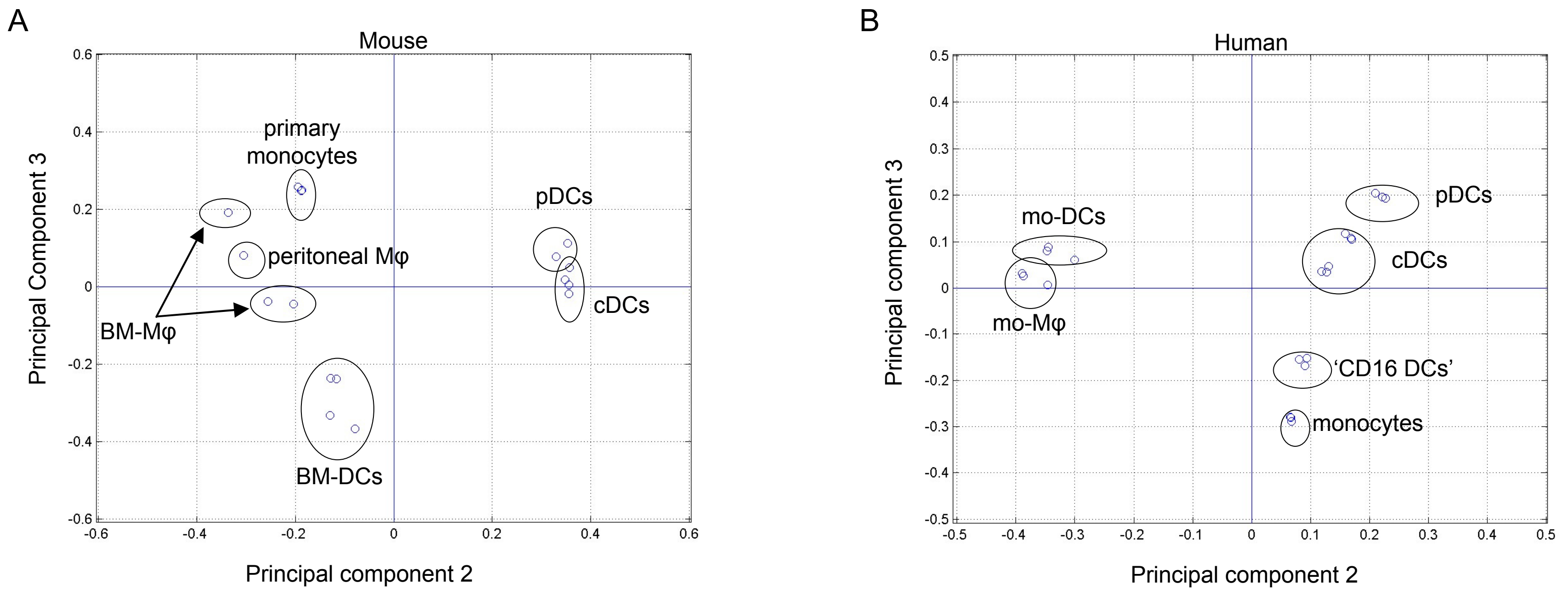

PCA analysis comparing LN-DCs, monocytes/macrophages and GM-CSF DCs in mouse (A) and human (B).

The data used for this analysis are . No gene filtering was performed prior to the analysis. The figure represents the second and third principal components, which accounted respectively for 7% and 3% for the mouse data and 5% and 2% for the human data. The first component accounted for 81% and 87% of the information for mouse and human datasets, respectively, and reflected the similarities among all samples.

BM-Mφ=bone marrow-derived macrophages; BM-DCs=bone marrow-derived GM-CSF DCs; mo-DCs= monocyte-derived GM-CSF DCs; mo-Mφ= monocyte-derived macrophages; pDCs=plasmacytoid DCs; cDCs= conventional DCs.

The datasets used are the same as those listed in Table 1 for figure 5A, with the exception for the mouse of the spleen monocytes corresponding to the GEO datasets GSM224733 et GSM224735.
