# Supplementary material for: Novel insights into the relationships between dendritic cell subsets in human and mouse revealed by genome-wide expression profiling
Source: Genome Biol. 2008 Jan 24;9(1):R17. doi: 10.1186/gb-2008-9-1-r17 (PMC2395256; doi:10.1186/gb-2008-9-1-r17)
Supplement: Additional file 7 — Real-time PCR data for the pattern of expression of 27 genes across mouse leukocyte subsets. [file gb-2008-9-1-r17-S7.pdf]

Gene signature	expt series	genes	Mono/Macro	pDC	CD8a cDC	CD11b cDC	NK	CD8 T	CD4 T	B	Splenocytes	Primers (F=Forward; R=Reverse)
non-DC												
	#	Gimap4	0.216	*	*	*	0.740	1.000	0.688	0.930	-	F-CGG GGT TCA TCC CAG AAA GTT; R-CCC CAA GGA TAC TGT TCC CTG
	#	Vps37b	0.603	*	*	*	0.601	1.000	0.504	0.491	-	F-ACA TGG TGG GGG TAT GGA; R-TTT GGG TCA GGC GAG CTT TC
	#	Fcer2a	*	*	*	*	*	*	*	1.000	-	F-CTG GGA ACC TCC TAG AAA GC G; R-CCC CAC CAA CAT GAG CTG T
	$	Lat	-	0.004	*	0.001	*	0.732	1.000	-	0.072	F-TCC CTG TTG TCT CCT CTG CT; R-CTC TGC GCT CTC CTC ATC CT
pan-DC												
	#	Flt3	0.008	0.985	1.000	0.844	*	*	*	*	-	F-GTG ACT GGC CCC CTG GAT ACG AG; R-TCC AAG GGC GGG TGT AAC TGA ACT
	#	Scarb1	0.209	0.831	1.000	0.806	*	*	*	*	-	F-TTT GGA GTG GTA GTA AAA AGG GC; F-TGA CAT CAG GGA CTC AGA TAG
	#	Sh3tc1	*	0.469	0.910	1.000	*	*	*	*	-	F-TGT ATC CAA CAG ACC TGA CC; R-GTC TTA AAT GTC ACC ACG ATC C
	#	Trit1	*	1.000	0.459	0.332	0.012	0.007	0.015	0.007	-	F-AAG TGG TTG ATC GGA AAG TAG AG; R-CTC ATC TAG AAC TGC CTG GTC
	$	Bri3bp	-	1.000	0.399	0.492	0.067	0.129	0.153	-	0.262	F-AGA GCG TGA GCA GCC TCT TC; R-CCA AAC TTT CCA TAA CGT CTC TAA GA
cDC												
	#	Ciita	0.011	0.002	0.755	1.000	*	*	*	0.014	-	F-ACA CAG ATA CCA TCA ACT GC; R-CCA ATG TGC TCT ATG AAG AGG
	#	Arhgap22	0.007	0.004	0.523	1.000	*	*	*	*	-	F-AGA TAC ATC TGC AAG TTT ATG G; R-GAT GGT TAC TGG ATC CTC TAT CTG
	#	Btbd4	0.041	0.084	1.000	0.622	*	*	*	*	-	F-ATC ACT TCT CAC TAC CGG CAT; R-ACG TTC TTA TGT GCC TTG AAG AC
	#	Slamf8	*	*	1.000	0.992	*	*	*	*	-	F-TCT CCT TCC CGT TGT GGT TG; R-CCA GAT AGC CTC ACG CAC TTG
	$	9130211I03Rik	-	0.004	1.000	0.841	*	*	*	-	0.015	F-CGG AAG AAG CAG ACC CAG AAG; R-CTG CGC AGC ACA GAG TTC TC
	$	Nav-1	-	0.007	0.052	1.000	*	*	*	-	0.046	F-CTC AAA GTC GCA GGC AAA CC; R-GAG CGT TGT AGC CCA GTG TTC C
CD8a cDC												
	#	Igsf4a	0.159	0.144	1.000	0.419	*	*	*	*	-	F-GAT CCC CAC AGG TGA TGG AC; R-TGA TGG TTG CCA CTT CTC CT T
	#	Gcet2	*	*	1.000	*	*	*	*	*	-	F-AGG ACAA CCA GAT ACT TCA GGG; R-AGG ATG TAG CAC AAC TCT TCT GT
	#	BC028528	*	*	1.000	*	*	*	*	*	-	F-TTG ACC TTT GTC CCA ATG C; R-AGG TTG GAG GCA CAC ATA GG
CD11b cDC												
	#	Pram1	*	*	*	1.000	*	*	*	*	-	F-CCC ATC AAT ATC CAA AGC TTC AG; R-ACA GCA TTT CAT CTC TGC CT
	#	Cyp4f16	*	*	*	1.000	*	*	*	*	-	F-GCT GAC ACC TTT ATG TTT GGA; R-AGG TCG TCC CAT TCA ATC TC
	#	Gpr43	*	*	0.001	1.000	*	*	*	*	-	F-GAT TAC AGG TTC CAC AGC AC; R-AGT TCT GAC TGC TCA CAT CC
pDC												
	#	SiglecH	*	1.000	*	*	*	*	*	*	-	F-GGA GGC AAA ACA TGG AAT TTA TG; R-CAC ATC ACA TTG GTA GGA CGA C
	#	Epha2	*	1.000	*	*	*	*	*	*	-	F-GGC CGA GCG CAT CTT TAT TG; R-AGT CAC TGC TGA CCG TGA TCT
	#	Pacsin1	*	1.000	*	*	*	*	*	*	-	F-GAG GTG GGG AAC TAC AAG CG; R-GTA TGC CTT CTC GAT CTT GGC
	$	Tex2	-	1.000	0.105	0.039	0.002	0.046	0.331	-	0.105	F-CCG GCG GGT GTT TCT G; R-GAC TGC TGT GCC TGC TGT GT
	$	Runx2	-	1.000	0.005	0.040	0.172	0.009	0.005	-	0.021	F-CCA GGC AGG TGC TTC AGA A; R-AGG GAT GAA ATG CTT GGG AAC T
	$	Tcf4	-	0.398	0.049	0.026	0.179	0.002	0.010	-	1.000	F-GGG CTC AGG GTA CGG AAC TAG; R-TCA GAC CCA CGC CAT C

* = NOT DETECTED

- = NOT TESTED

Samples were run on an ABI 7900HT Fast Real-Time PCR System (Applied Biosystems), 40 cycles, using the QuantiTect SYBR Green PCR Kit (Qiagen)

Relative gene expression was calculated using the ΔΔCt method

TATA box binding protein (TBP) and Hypoxantine-guanine phosphoribosyltransferase (Hprt) were used as the endogenous control housekeeping genes

For each gene, the value for the cell type with highest expression was calculated as 1

experiment series 1 with TBP housekeeping control gene (SHR)

$ experiment series 2 with Hprt housekeeping control gene (GB)

Primers for TBP	F-AGAACAATCCAGACTAGCAGCA	Primers for Hprt	F-GGC CCT CTG TGT GCT CAA G
	R-GGGAACTTCACATCACAGCTC		R-CTG ATA AAA TCT ACA GTC ATA GGA ATG GA
