# Supplementary material for: Novel insights into the relationships between dendritic cell subsets in human and mouse revealed by genome-wide expression profiling
Source: Genome Biol. 2008 Jan 24;9(1):R17. doi: 10.1186/gb-2008-9-1-r17 (PMC2395256; doi:10.1186/gb-2008-9-1-r17)
Supplement: Additional file 8 — PACSIN1 expression in human pDCs versus PBMCs by RT-PCR and western blotting. [file gb-2008-9-1-r17-S8.pdf]

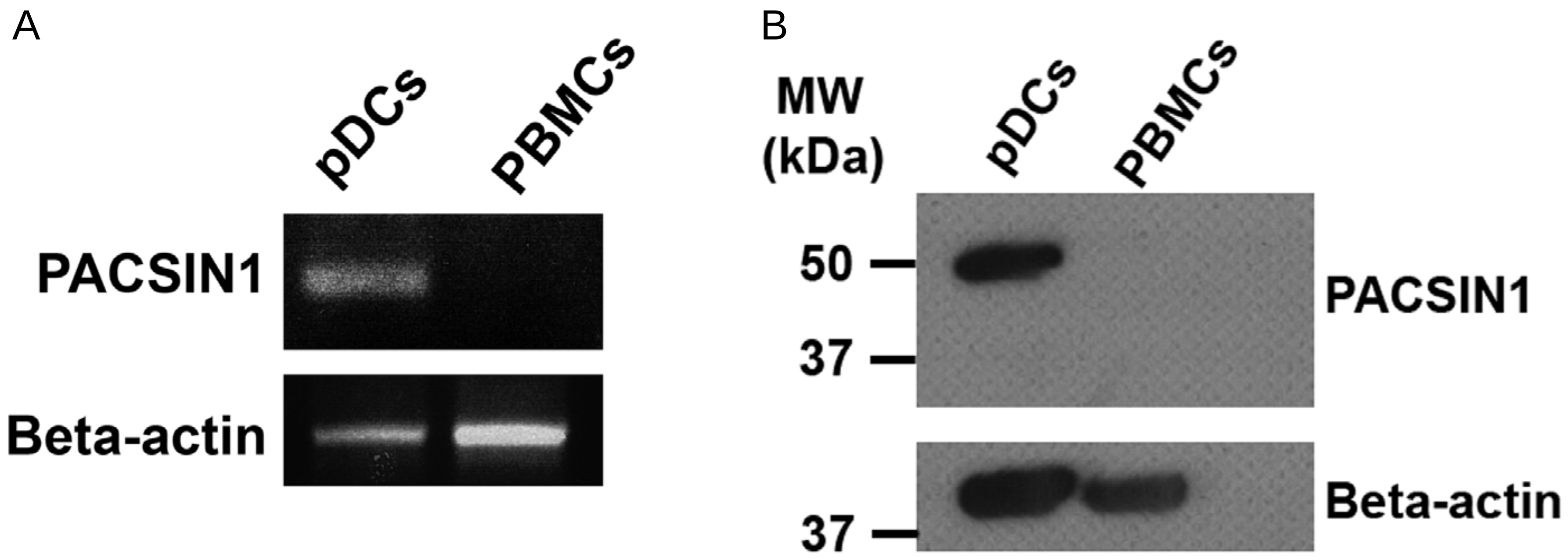

Selective expression of PACSIN1 in human pDCs.
pDCs (>95% purity) were isolated from PBMCs using magnetic selection of CD304 / BDCA-4^{+} cells (CD304 Microbead kit, MILTENYI).
A) RT-PCR analysis of the expression of *PACSIN1* mRNA in human pDCs versus PBMCs. Total RNA was extracted from pDCs versus PBMCs of the same donor using the RNeasy Mini kit (QIAGEN). RT-PCR was then performed using the following primers FWD-5'-CATCCATGTGTACCGTGAGC and RSE-5'-AGGAAGGTCTGGGTTCCACT, and an annealing temperature of 60°C. Beta-actin was amplified as a houskeeping control gene, using the following primers FWD-5'-ATCTGGCACCACACCTTCTACAATGAGCTGCG and RSE-5'-CGTCATACTCCTGCTTGCTGATCCACATCTGC.
B) Western-blot analysis of the expression of PACSIN1 in human pDCs versus PBMCs. Standard western blot procedures were used to evaluate PACSIN1 levels in whole lysates of human pDCs versus PBMCs, using an antibody specific for this molecule (a generous gift from Dr. Markus Plomann, University of Cologne, Germany). Beta-actin expression levels were assessed in the same samples as a houskeeping control protein.
